# The SynMuvA *lin-15A* licenses natural transdifferentiation by antagonizing identity safeguarding mechanisms

**DOI:** 10.1371/journal.pgen.1012290

**Published:** 2026-09-25

**Authors:** Sarah Frieda Becker, Marie-Charlotte Morin, Julien Lambert, Shashi Kumar Suman, Francesco Nicola Carelli, Alex Appert, Stéphane Roth, Sarah Hoff-Yoessle, Jessica D. Medina-Sanchez, Manuela Portoso, Julie Ahringer, Sophie Jarriault

**Affiliations:** 1 Department of Development and Stem Cells, Institut de Génétique et de Biologie Moléculaire et Cellulaire (IGBMC), CNRS UMR 7104, INSERM U1298, Université de Strasbourg, Illkirch, France; 2 Current address: Biozentrum, University of Basel, Basel, Switzerland; 3 The Gurdon Institute and Department of Genetics, University of Cambridge, Cambridge, United Kingdom; 4 Current address: Institute of Pharmaceutical Sciences, ETH Zurich, Zurich, Switzerland; 5 Institut Curie, INSERM U934/CNRS UMR 3215, Paris Sciences et Lettres Research University, Sorbonne University, Paris, France; Aix-Marseille Université: Aix-Marseille Universite, FRANCE

## Abstract

The mechanisms that restrict or enable latent cellular plasticity have attracted growing interest over the past decade, with important implications for cancer and regenerative therapies. However, the diversity of both pro- and anti-plasticity mechanisms remains incompletely understood. Here, we identify the THAP domain gene *lin-15A* as a novel factor involved in the natural rectal-to-neuronal Y-to-PDA transdifferentiation in *Caenorhabditis elegans*. Our genetic analyses show that *lin-15A* acts in parallel to previously described plasticity factors. Null mutant suggests that, rather than driving the process, LIN-15A licenses its initiation by antagonizing several chromatin-modifying complexes known to safeguard differentiated cell identities. In addition, our data show that even cells developmentally programmed to undergo transdifferentiation exhibit reprogramming barriers. Interestingly, *lin-15A* is not a core plasticity factor per se but acts as one specifically in the Y cell context. Together, our findings support a model in which diverse molecular activities coordinate controlled cell identity conversions: plasticity factors function as Drivers, while others like *lin-15A*, termed here transdifferentiation Licensers, attenuate identity safeguarding mechanisms, thereby facilitating transdifferentiation.

## Introduction

Over 60 years ago, John Gurdon demonstrated that nuclei of differentiated, somatic cells can be reprogrammed and allow the development of a frog, when inserted into an enucleated frog egg [1,2]. Since then, numerous studies have shown that transcription factor-based inducing cues, ranging from only one to a small cocktail of TFs, could trigger the reprogramming of various differentiated cells into ES-like cells, as well as into another differentiated identity (aka induced direct reprogramming or induced transdifferentiation), both *in vitro* [3,4] and *in vivo* [5,6]. The control and plasticity of differentiated cell identities have emerged as central topics in biomedical research, particularly in the contexts of cancer, tissue regeneration, and cellular reprogramming [7,8]. Understanding how fully differentiated cells can shift their identity, and why not all cells manage to do so, offers valuable insights into developmental biology, disease progression, and therapeutic innovation.

In the past few decades, extensive research using induced reprogramming systems has highlighted several roadblocks. For instance, induced direct reprogramming typically exhibits low efficiency, with only a small fraction of cells successfully converting, and this efficiency varies widely between cell types, suggesting the presence of intrinsic barriers. In addition, induced direct reprogramming is more easily achieved by over-expressing specific transcription factors in cells that are not fully differentiated compared to those that are differentiated [4,6]. Moreover, conversion is often incomplete, with cells retaining an epigenetic memory or original lineage gene expression, or yields immature cells. These observations have led to the concept of reprogramming barriers limiting the latent plasticity of differentiated cells.

A number of mechanisms have been proposed to explain the resistance of most differentiated cells to induced reprogramming. Extra-cellular matrix properties [9], cell signaling pathways [10], cell identity acquisition and maintenance transcription factors (TFs) [11], fate-stabilizing TFs [12–14], as well as genome organization, including 3D chromatin architecture, histone modifications and chromatin remodelers [4,15], have all been proposed to modulate the response of differentiated cells to reprogramming cues. These mechanisms may altogether underlie the active safeguarding of the differentiated identities suggested by Blau and Baltimore [16].

Do barriers exist in cells that naturally undergo direct reprogramming? What would these barriers be and how are they removed? Natural instances of direct reprogramming, in which a differentiated cell acquires itself or gives rise to a daughter cell with a different differentiated identity without a passage through a stable intermediate, are not very frequent in a given species. In *C. elegans*, although some cells naturally change their identity during development, the majority retain their identity. In other model systems as well, including vertebrates, most cell do not naturally change their identity apart from some specific developmental, regenerative or pathological contexts. However, by contrast with induced direct reprogramming, natural direct reprogramming (aka natural transdifferentiation, or Td), is robust and yields a stable and mature final cell [17,18]. Thus, natural Td presents a compelling model of endogenous cell fate flexibility operating outside of artificial manipulation [19–26].

Here, we have used an instance of natural Td in *Caenorhabditis elegans* to address these questions. Given its transparency, complete toolbox for genetical approaches and fully resolved stereotypical somatic lineages [27] it is possible to unambiguously examine cellular identities at the single cell level in *C. elegans*, making it a prime model for the study of natural Td [6,28–32]. The transdifferentiation of the rectal Y cell into the cholinergic motorneuron PDA in the hermaphrodite was the first evidence of natural Td in the worm and is the best characterized event to this day ([28]; reviewed in [33]) (Fig 1A). This specialized epithelial cell is born during embryogenesis and forms the rectal tube together with 5 other rectal cells: B, U, F, K and K’ that share similar morphology and markers [27]. At the end of the L1 larval stage however, the rectal Y retracts from the rectum (Td initiation), dedifferentiates very transiently into an unipotent intermediate, and re-differentiates into the PDA neuron in a stereotypical and robust process [17,34]. Y-to-PDA occurs directly and without a cell division [28,35]. The erasure of the initial rectal identity requires the action of conserved NODE (Nanog and Oct4-associated deacetylase)-like complex [36] while the H3K4me3 histone methyltransferase SET-2/SET1 ensures Td robustness in face of environmental stresses [17]. The NODE-like complex, composed of SEM-4/SALL4, CEH-6/OCT, EGL-27/MTA1 acts together with the region-specific HOX TF EGL-5, the conserved plasticity factor SOX-2/SOX2 and the bHLH transcription factor HLH-16 [37,38], as the Drivers of the initiation step [36]. The loss of any of these factors results in the failure of Td initiation: no retraction of Y from the rectum and maintenance of the initial rectal identity. ZTF-11/Myt1 is also required for this step, however its relation to the Drivers is still unknown [13]. Re-differentiation into PDA is dependent on UNC-3/EBF [34] acting as a terminal selector, and facilitated by the histone demethylase JMJD-3.1 [17]. While the NODE-like complex and other drivers of Y-to-PDA initiation have been identified, the full repertoire of factors controlling this process—and their mechanisms of action—remains incomplete.

**Fig 1:**
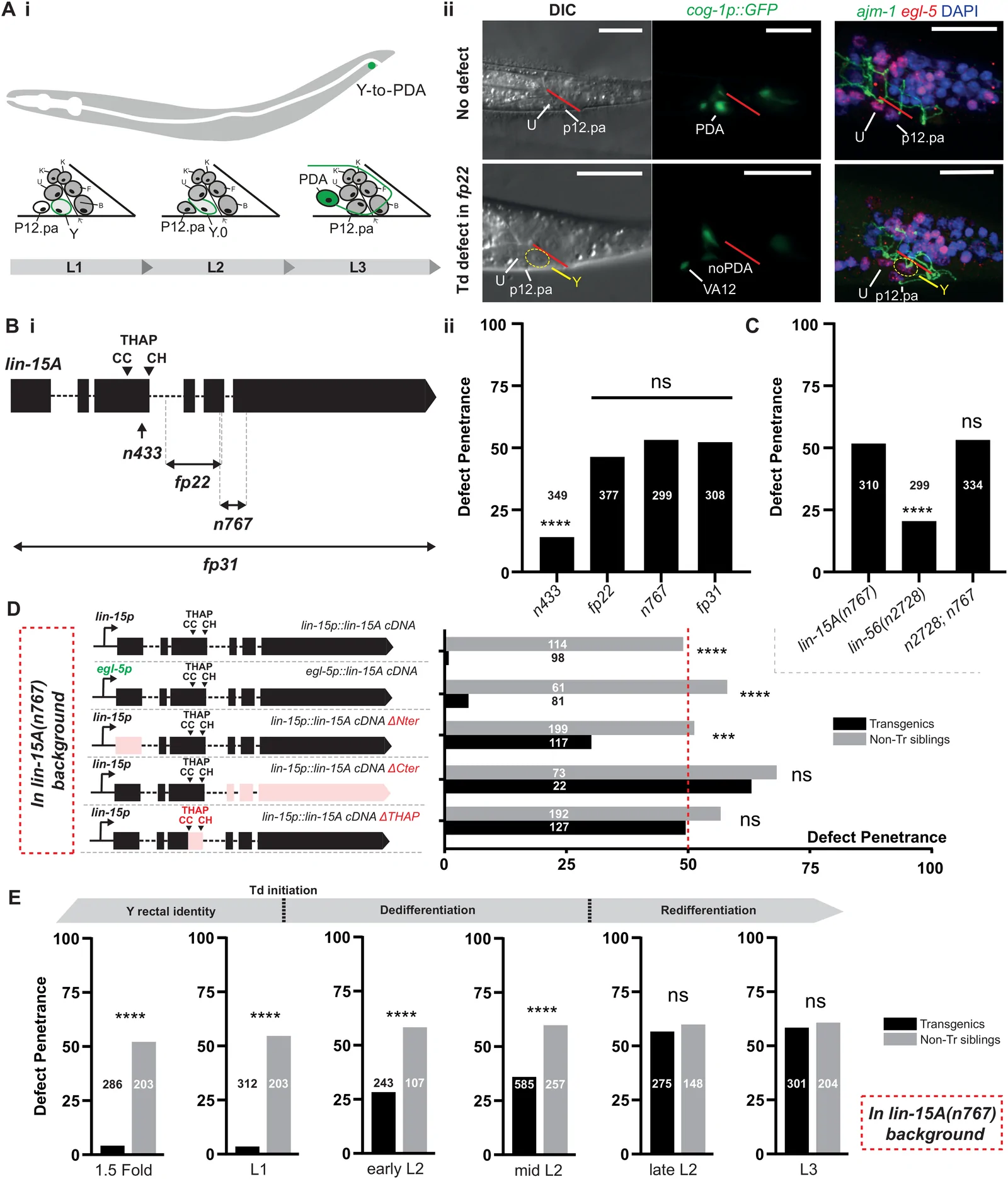
LIN-15A and LIN-56 are two novel factors required for Y-to-PDA initiation. **A i** Y-to-PDA transdifferentiation (Td) and typical defects of *fp22* mutants. At the end of L1 stage, Y retracts from the rectum and dedifferentiates. PDA redifferentiation takes place during the L2 and late L3 stages. **ii** Defects induced by *fp22* (bottom panels) result in the inability of the Y cell to retract from the rectum and initiate its Td. As a result, 3 epithelial nuclei (Y, P12.pa and U) are visible by DIC in the anterior rectum of L4 worms, instead of 2 (left panels), and the Y cell retains its rectal-epithelial features (apical junctions’ component AJM-1) and markers (EGL-5; red). Consequently, no PDA is made (middle panels). Images of the rectal area, the rectal slit is indicated in red. Anterior to the left and ventral to the bottom. Scalebars = 10 *μ*m. **B i** Alleles of *lin-15A* used in this study, and **ii** their respective defect penetrance: *fp22*, *fp31 and n767* are null alleles and induce a 50% defect penetrance in Td initiation. Weaker *n433* causes defects at a penetrance of 15%. X2 and Fisher’s exact tests with Bonferroni corrections for proportion comparisons. In all barplots in this study, the numbers associated with the histograms or scatter plots indicate the total number of animals (n) scored in the cohort. **C** *lin-15A* and *lin-56* act in cooperation for Y-to-PDA Td. X2 and Fisher’s exact tests with Bonferroni corrections for proportion comparisons. **D** Rectal-specific rescue of *lin-15A* by *egl-5* promoter fully suppresses the transdifferentiation defects demonstrating that *lin-15A* activity in the rectal cells is sufficient for its role in Y-to-PDA Td. The C-term and THAP domain of LIN-15A are critical for its role in Y-to-PDA Td. Its N-term domain is less critical. Rescuing constructs are represented on the left side. Experiments are carried out in *lin-15A(n767)* background, with the standard 50% defect penetrance of this mutant indicated by the vertical red dotted line. Non-transgenic siblings (grey bars) were scored in parallel, and defect penetrance represents the absence of a PDA neuron. Fisher’s exact tests with Bonferroni corrections for proportion comparisons. **E** Timing of LIN-15A requirement in Y-to-PDA transdifferentiation using time-controlled rescue experiments with a heat-shock inducible promoter. Providing LIN-15A activity only in the L1 stage is sufficient to rescue *lin-15A* mutant Td defect. LIN-15A rescue during the dedifferentiation step (L2 stage) partially suppresses the defects highlighting a possible timeframe of initiation spanning until the mid-L2 stage. Providing LIN-15A activity after the initiation step has no effect (late L2 and L3 stages). Experiment carried out in *lin-15A(n767)* background. Fisher’s exact tests with Bonferroni corrections for proportion comparisons.

In this study, we identify the SynMuvAs LIN-15A and LIN-56 as novel players in Y-to-PDA initiation, acting cooperatively. *lin-15A* is part of the *lin-15* operon together with the SynMuvB gene *lin-15B* [39,40] and both *lin-15A* and *lin-56* code for a THAP domain ZnF nuclear protein whose function is unknown [41]. We report that LIN-15A binds naked and nucleosome-bound chromatin, that its action in Td occurs in the rectal cells and requires its THAP domain. Our genetic interaction studies demonstrate that LIN-15A and LIN-56 function in a parallel pathway to the factors of the NODE-like complex. We also show that LIN-15A and LIN-56 are similarly required for Y-to-PDA in males (which occurs through Y division), but that contrary to the NODE-like complex, LIN-15A is not involved in the other rectal-to-neuronal transdifferentiation K-to-DVB [32], and that it can even restrict cellular plasticity in the embryo. Hence, LIN-15A acts as a plasticity factor in specific cell contexts, like that of the Y cell, whether in presence (males) or in absence (hermaphrodites) of a cell division. In contrast to VPC specification, we show that the role of LIN-15A is independent of *lin-3/EGF* during Td. However, we found that it antagonizes most of the SynMuvBs chromatin factors and complexes, like the DREAM complex, that negatively regulate chromatin activity, suggesting a role in the control of gene expression. SynMuvB factors appear to act as brakes for Y-to-PDA transdifferentiation, likely by safeguarding the initial rectal identity. Last, our ChIP-seq data provide evidence consistent with a chromatin-based mechanism underlying the genetic antagonism between LIN-15A and SynMuvB factors, with loss of LIN-15A being associated with increased DREAM occupancy at a subset of loci.

## Results

### Two novel factors required for Y-to-PDA transdifferentiation: LIN-15A and LIN-56

Via an unbiased EMS screen for mutants inducing defects in Y-to-PDA transdifferentiation (Td) (PDA neuron not produced), we retrieved the allele *fp22* which leads to approximatively 50% of the animals failing to produce PDA (Fig 1Aii and 1B). EMS-density mapping using WGS analysis [42] identified *fp22* to be a 502 bp deletion in the coding sequence of the SynMuvA gene *lin-15A* (Fig 1B). The two canonical alleles of *lin-15A*, *n433* (weak loss of function) and *n767* (a 178 bp deletion-insertion in the C-term domain of LIN-15A, supposed null [41]) equally result in Y-to-PDA defects with a penetrance of respectively 15% and 50% (Fig 1B), confirming that *fp22* is a novel *lin-15A* allele (Fig 1B and 1D). Although *n767* behaves as a null allele, the molecular lesion does not entirely eliminate *lin-15A* but results in a truncated protein, leaving the possibility that the 50% defect penetrance observed could be due to residual *lin-15A* activity. We thus generated *fp31*: a deletion of the entire *lin-15A* gene (keeping *lin-15B* unaffected). As for *fp22* and *n767*, *fp31* resulted in a 50% penetrant Y-to-PDA defects (Fig 1B). Genetic complementation tests further validated that *fp22* and *n767* are null alleles of *lin-15A* (S1A Fig). For the rest of this study, experiments were conducted with the canonical *n767* allele unless otherwise stated.

LIN-15A was previously described to physically interact and cooperate with LIN-56, which also possesses a THAP-like C2CH domain [41], in the repression of *lin-3/EGF* expression during VPC specification [39,40]. Therefore, we tested whether a similar interaction occurs during Y-to-PDA. We found a 20% penetrance of Y-to-PDA defects in *lin-56(n2728)* null mutants [41]. Double mutants *lin-56(n2728); lin-15A(n767)* displayed the same defect penetrance of 50% than single *lin-15A(n767)* mutants (Fig 1C) indicating that the two factors act in the same pathway during Y-to-PDA. The lower penetrance of *lin-56* null mutants likely reflects the fact that it functions as an accessory factor whereas LIN-15A constitutes the critical regulator.

Other SynMuvA genes, in addition to LIN-15A and LIN-56, repress the expression of *lin-3/EGF* during VPC specification [43]. Thus, we tested the putative involvement of other SynMuvAs in Y-to-PDA. However, none of the other tested SynMuvA appears to be required for Y-to-PDA (S1B Fig).

Together, these results demonstrate the requirement of two novel factors in Y-to-PDA transdifferentiation: LIN-15A and LIN-56, where, contrary to VPC specification, they act independently of other SynMuvAs.

### LIN-15A acts through its THAP-domain, at the time of Y-to-PDA initiation, in the rectal cells

A defect in Y-to-PDA could result by a failure in the initiation step, where Y is retracted from the rectum, dedifferentiates and loses its epithelial features, or the re-differentiation step during which it becomes PDA and acquires neuronal features [28,34]. DIC images of *lin-15A(fp22)* L3 and older worms (when Td should have taken place already) showed that the Y cell is retained in the rectum with apparent rectal morphology, a class I defect [34], indicating a Td initiation defect (Fig 1Aii). Immunostainings against the epithelial and rectal markers, AJM-1 and EGL-5, respectively, confirmed that Y still carries rectal epithelial features and remains on the anterior side of the rectum, beyond the time point of Td initiation in Y-to-PDA defective worms (9/9 Y-to-PDA defective out of 21 animals scored) (Fig 1Ai). Live reporters for additional rectal-epithelial markers showed that the persistent Y cell retains a rectal identity and that expression of these rectal markers was unaltered prior to Td initiation (S1C Fig). Hence, LIN-15A is required for the initiation step. Of note, LIN-15A is expressed in Y prior to Td initiation (S1D and S1E Fig), consistent with a role in promoting the identity switch.

Since *lin-15A* is broadly expressed, including in the rectal cells, during the earlier stages of *C. elegans* development (S1E Fig), we sought to assess in which tissues and at what stage it was required for Td. Expression of *lin-15A* in the rectal cells using the *egl-5p* promoter rescued the transdifferentiation defects to the same extent as expression driven by the endogenous *lin-15* promoter (Figs 1D and S1D). While the absence of a Y-specific promoter precludes from formally distinguishing between Y-cell autonomous and non-autonomous functions, these experiments demonstrate that *lin-15A* acts within the rectal cells during Y-to-PDA.

We further found that both the C-terminal domain as well as the C2CH THAP domain are necessary for the function of LIN-15A for Td (Figs 1D and S1D). In contrast, we found that the N-terminal domain was only partially required since expressing a N-terminal truncated *lin-15A* cDNA could only partially rescue the defects. The requirement of the THAP domain suggests a nucleic acid binding role for LIN-15A in Y-to-PDA. In accordance with this hypothesis, CRISPR/Cas9 mCherry-tagged LIN-15A localizes in the nucleus, and purified LIN-15A is able to bind naked and nucleosome loaded DNA (S1E and S6 Figs).

We then assessed when LIN-15A activity was required for Y-to-PDA initiation. Y is born around 300 minutes after fertilization (during embryogenesis; [44]), and Y-to-PDA initiation only occurs at the end of the first larval stage [28]. Hence LIN-15A activity could be required at any time during that period as it is constantly expressed in the rectal cells until the transdifferentiation (S1E Fig). Using a heat-shock (hs) inducible *lin-15A cDNA* transgene, we found that providing LIN-15A activity at the end of the L1 stage is sufficient for a WT Td, suggesting that *lin-15A* acts around Y-to-PDA Td initiation (Fig 1E). Indeed, as for a hs during embryogenesis, a late hs-mediated rescue in L1 is sufficient to fully suppress the defects. Rescuing LIN-15A activity after Y-to-PDA completion (in L3) on the other hand had no effect. Interestingly, a partial rescue could still be observed until the mid L2 stage. These could be indicative of a time-window in Td initiation: even though it starts just after the L1 stage, the process could still be rescued in a slightly larger time window by LIN-15A expression over the next hours.

### Two parallel initiation pathways

We previously reported that Y-to-PDA initiation was promoted by a plasticity cassette composed of a NODE-like complex - CEH-6/OCT, SOX-2/ SOX, SEM-4/SALL4, EGL-27/MTA1 - the EGL-5 Hox and the bHLH HLH-16 transcription factors [36–38]. As LIN-15A is equally required for the initiation step, we wondered whether it was acting in the same pathway as these factors.

First, we tested if *lin-15A* expression is under the control of the plasticity cassette. We found that endogenously tagged LIN-15A expression was unchanged in *sem-4(n1971)*, *egl-5(n945)*, *egl-27(ok1670)* or *hlh-16(fp12)* mutants compared to WT animals (Fig 2A). These data suggest that *lin-15A* expression is not controlled by the previously described factors.

**Fig 2:**
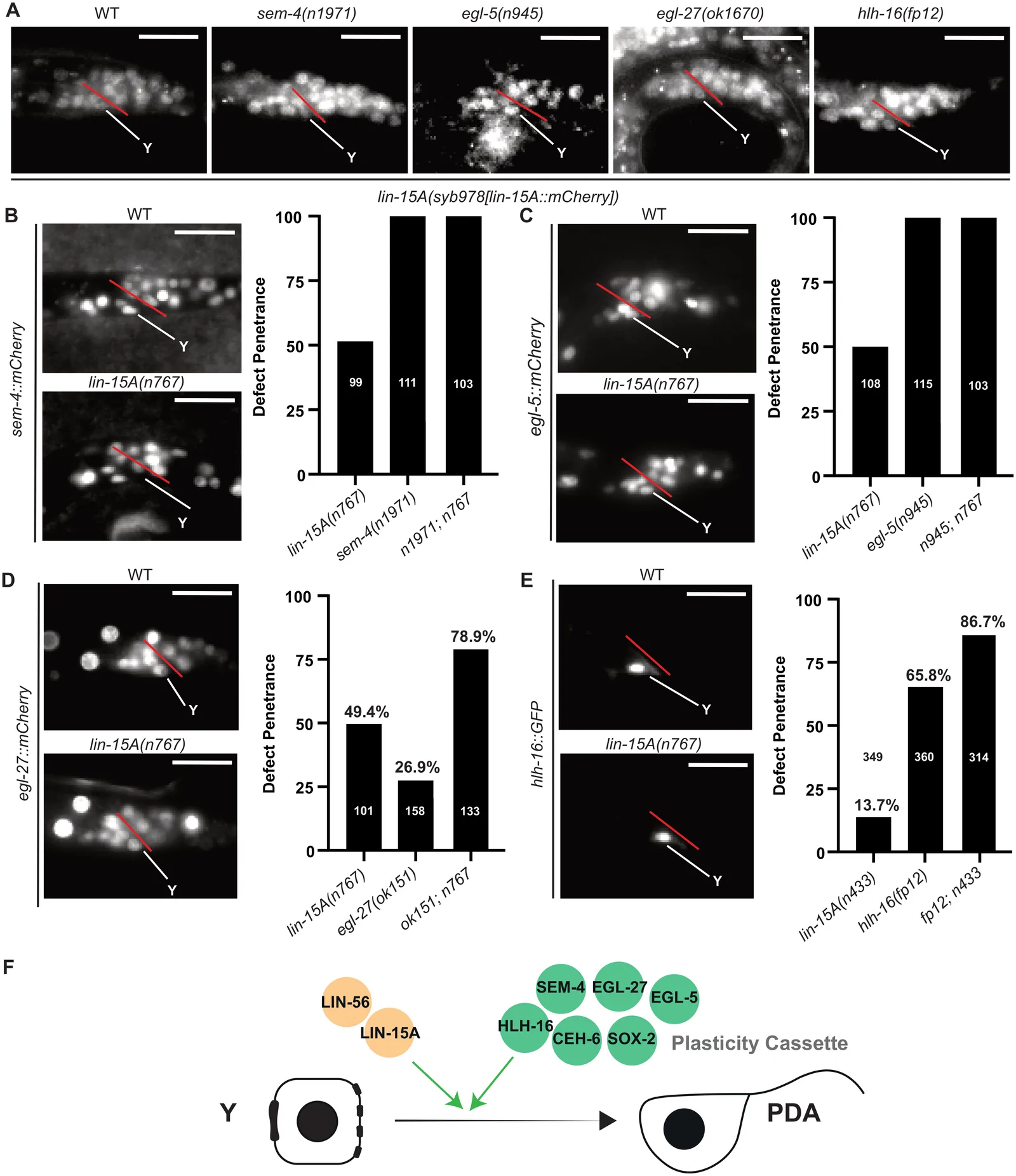
Two parallel pathways are required for Y-to-PDA initiation. All pictures: representative images of the rectal area during the L1 stage, prior to Y-to-PDA initiation. The rectal slit is indicated in red. Scalebars = 10 *μ*m. **A** Expression of endogenous LIN-15A (crispr Kin allele *syb978*) is not affected by the loss of any of the previously described factors *sem-4* (n = 50), *egl-5* (n = 47), *egl-27* (n = 50) nor *hlh-16* (n = 50) (for WT n = 50). **B and C** LIN-15A acts independently of SEM-4 (B) and EGL-5 (C): SEM-4 and EGL-5 expression are not affected by the loss of *lin-15A* (for SEM-4 WT, n = 51 / *lin-15A(n767)*, n = 48 – for EGL-5 WT, n = 52 / *lin-15A(n767)*, n = 47). Td defects induced by the loss of *sem-4* or *egl-5* are unchanged in double mutants with *lin-15A*. **D** LIN-15A acts independently of EGL-27: EGL-27 expression is not affected by the loss of *lin-15A* (WT, n = 48 / / *lin-15A(n767)*, n = 50). *lin-15A* null mutant induces defects in an additive manner when combined with weak loss of function *egl-27(ok151).* Likely-hood Ratio Test p-value (two-sided) between the *lin-15A* single and double mutants = 0.0034**. E**. LIN-15A acts likely independently of HLH-16: HLH-16 expression is not affected by the loss of *lin-15A* (WT, n = 50 / *lin-15A(n767)*, n = 47). *lin-15A(n433) (lf)* induces defects in an additive or slightly synergistic manner with strong loss of function *hlh-16(fp22).* LRT p-value (two-sided) between the *hlh-16* single and double mutants < 1 × 10^–9^. **F** LIN-15A and LIN-56 (yellow) act in parallel to Driver activities: the Plasticity Cassette NODE-like complex, SOX-2 and EGL-5 (green).

Next, we tested whether LIN-15A could activate these factors. We found that the expressions of fluorescent reporters for SEM-4, EGL-5, EGL-27, HLH-16, CEH-6 and SOX-2 remain unchanged in *lin-15A(n767)* mutants compared to WT animals (Figs 2B and 2E and S2). We next used genetic interaction tests to assess the relationship between *lin-15A* and these factors. As expected, the 100% penetrance of *sem-4(n1971)* and *egl-5(n945)* mutants remained unchanged in double mutants with *lin-15A(n767)* (Fig 2B and 2C). Double mutants between *lin-15A* and *egl-27* or *hlh-16* mutants that do not cause a full penetrance of Td initiation defects [36,37,42] showed additive effects, suggesting they act in different pathways (Fig 2D and 2E).

Together, our results show that LIN-15A and LIN-56 act in a distinct, novel pathway parallel to the plasticity cassette and therefore that multiple activities are required for successful Td initiation *in vivo* (Fig 2F): on the one hand, conserved plasticity factors (CEH-6/OCT, SOX-2/SOX, SEM-4/SALL4, EGL-27/MTA1, EGL-5/HOX and HLH-16/bHLH, altogether termed “Drivers”) drive dedifferentiation, and their loss results in complete failure of Td initiation; on the other hand, LIN-15A and LIN-56 facilitate the process, and their complete loss results in a lower penetrance of Td initiation failure.

### LIN-15A acts as a (pro-)plasticity factor specifically in Y

We next tested if *lin-15A* is necessary in other plasticity events in *C. elegans*. We first tested whether it was required for the K-to-DVB transdifferentiation [32] (Fig 3Ai). K is another rectal cell that undergoes a transdifferentiation to produce a motor neuron named DVB which is critical for the proper defecation motor program (DMP) of the animal [45–47]. In contrast to Y-to-PDA, K-to-DVB occurs through a cell division: K divides and its posterior epithelial daughter redifferentiates into DVB while the anterior daughter retains the rectal-epithelial identity. Nonetheless, the two Td exhibit similarities. Both Y and K are rectal cells and initiate their transdifferentiation at the end of the L1 stage. CEH-6/OCT, SOX-2/SOX2, SEM-4/SALL4 and EGL-5/HOX are also required for K-to-DVB, similarly to erase the rectal identity [32]. Interestingly, LIN-15A is also expressed in K in L1 (Fig 3Aii) which further raised the question of its possible involvement in K-to-DVB. We found that neither LIN-15A nor LIN-56 were required for K-to-DVB (Fig 3B).

**Fig 3:**
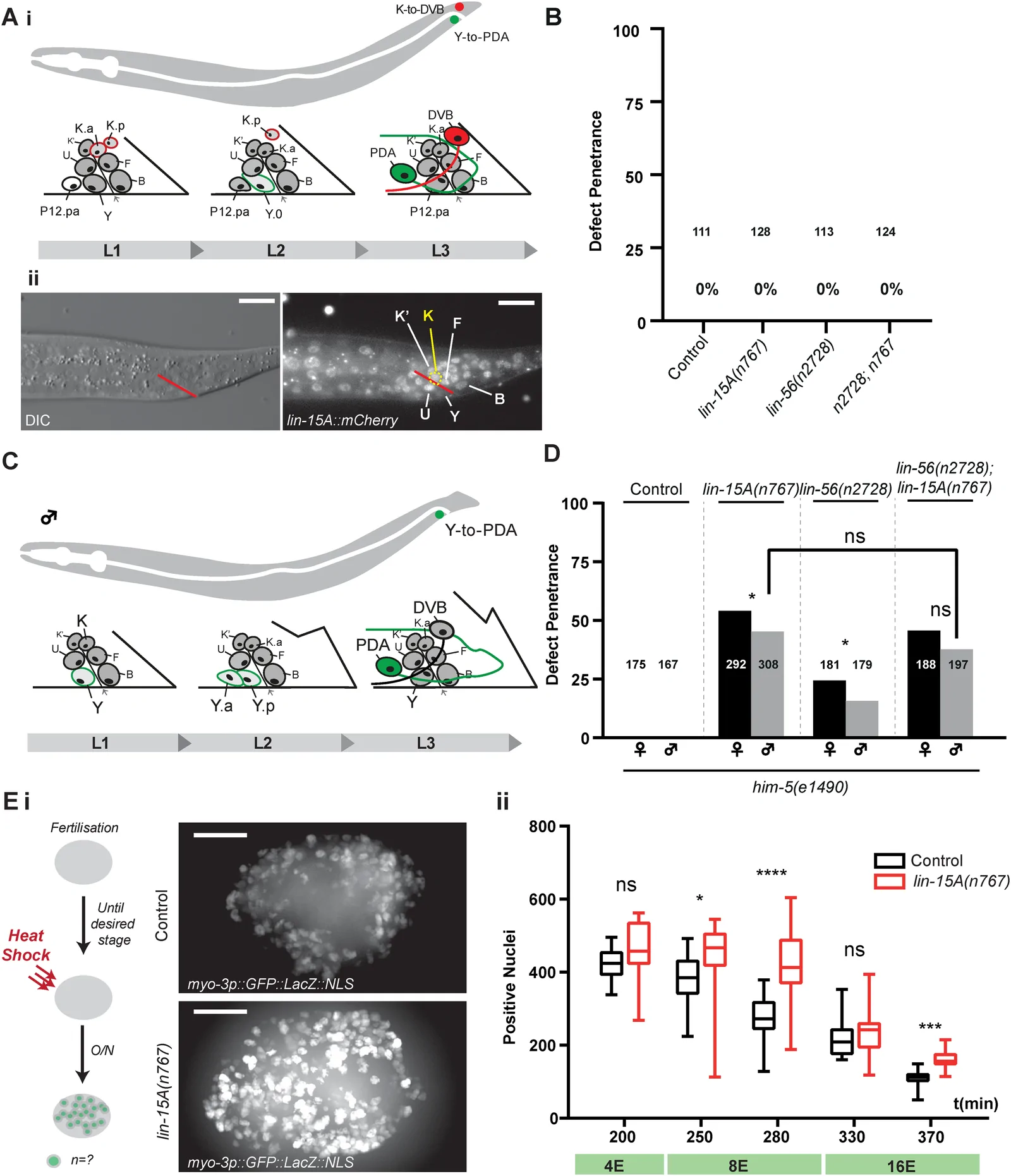
LIN-15A acts as a plasticity factor specifically in Y. **A i** K-to-DVB transdifferentiation. At the end of the L1 stage, the K rectal cell divides. During the L2 stage the posterior daughter K.p redifferentiates into the DVB motor neuron. **ii** LIN-15A is expressed in K (yellow) prior to its division. Representative images of the rectal area in L1 – prior to K-to-DVB Td. The different rectal cells are indicated, as the rectal slit in red. Scalebars = 5 *μ*m. **B** *lin-15A* loss does not induce K-to-DVB Td defects as revealed by the absence of a DVB motoneuron. **C** Y-to-PDA transdifferentiation in males. At the late L1 – early L2 stage, the Y cell divides. At the late L2 / early L3 stage, the anterior daughter Y.a redifferentiates into the PDA motoneuron. **D** LIN-15A and LIN-56 act in cooperative manner during Y-to-PDA Td in males as they do in hermaphrodites. The absence of a PDA neuron was scored in hermaphrodite (black bars) and male (grey bars) siblings from the same parental population and the same plates. Fisher’s exact tests with Bonferroni corrections for proportion comparisons. **E i** Representative images of heat-shocked WT (upper picture) or *lin-15A* mutant (lower picture) embryos displaying supernumerary *myo-3* + blastomeres when HLH-1/MyoD is induced at 200, 280 or 370 minutes post-fertilization. To control that variations in the number of fluorescent nuclei was not caused by variations in the total number of nuclei, the total number of nuclei was monitored and found to remain constant in the different conditions. Examples of the z-stacks used for the 3D reconstruction and converted nuclei scorings are available at: https://drive.google.com/drive/u/1/folders/1TSElEi42NpMuBWCJ-KMi14RGqW4d0wFG **ii**. Number of converted blastomeres (*myo-3*-positive nuclei) after heatshock (hs) at the five timepoints in control (black) and *lin-15A(n767)* animals (red) as observed at birth (the number of embryos scored for each hs time point is as follows: 200min wt = 19 *n767* = 10 - 250min wt = 21 *n767* = 20 - 280min wt = 25 *n767* = 17 - 330min wt = 20 *n767* = 19 - 370min wt = 25 *n767 = 16*). In non-heatshocked WT animal, the number of body-wall muscle nuclei at birth is 95. Mann-Whitney tests with Bonferroni corrections for rank comparisons.

As K-to-DVB involves a cell division, we wondered whether LIN-15A would be required in Y-to-PDA because of the lack of cell division. We therefore tested its involvement in Y-to-PDA in males, which occurs through a cell division (Riva C. and Jarriault S. *pers. comm*) (Fig 3C). Using the mutant *him-5(e1490)* which naturally produces around 35% of males within a population, we were able to score the presence or absence of PDA in males and hermaphrodites from the same mothers. We found that both LIN-15A and LIN-56 are required for Y-to-PDA in the males, and in the same non-additive and non-synergistic manner as in hermaphrodites (Fig 3D). Hence, the role of LIN-15A does not seem related to the absence of cell division, but rather appears specific to the Y-to-PDA transdifferentiation context. We next examined whether it promoted early blastomere plasticity, especially as LIN-15A is widely expressed during *C. elegans* embryonic development (S1E Fig) and is progressively lost as development advances. To test this hypothesis, we assessed whether loss of *lin-15A* impacts the Multipotency-to-Commitment Transition (MCT, reviewed in [6]) which we found, using an improved protocol, to occur progressively from 200 to 370 minutes after fertilization (Lambert and Jarriault, *in prep*). We reasoned that if LIN-15A promoted embryonic cellular plasticity, the MCT should occur earlier in *lin-15A* mutants. We therefore performed a series of cell-fate challenge assays by forcing the expression of the muscle determinant HLH-1/MyoD, known to transform early embryos into a ball of muscle cells when expression is forced before the MCT [48–50]. HLH-1/MyoD expression was overexpressed at 5 different time points during the timing of the progressive MCT (200/250/280/330/370 minutes post-fertilization) using a heat-shock promoter (Lambert and Jarriault, *in prep*). The strains used for the cell fate challenge assays carry an integrated array expressing an NLS::GFP under the control of the body-wall muscle-specific promoter *myo-3p*. Therefore, any cell differentiated into the body-wall muscle identity (both naturally and transdetermined) displays strong GFP signal (Fig 3E). Our results show that absence of *lin-15A* does not result in an earlier MCT, as would have been expected if it promoted early blastomere plasticity. Surprisingly, we even found that the loss of *lin-15A* increased the number of converted nuclei at 250 (early 8E stage), 280 (late 8E stage) and 370 (late 16E stage) minutes post-fertilization (Fig 3E). In the control strain, the MCT clearly began between 200 and 250 minutes. In *lin-15A(n767)* mutants however, it is only first seen between 280 and 330 minutes. Overall, *lin-15A(n767)* blastomeres can be transdetermined for a longer period of time than their WT counterparts. Hence LIN-15A restricts the plasticity of the blastomeres rather than increasing it.

Together, these results highlight that LIN-15A does not increase cellular plasticity in general but acts as a pro-plasticity factor in specific cellular contexts like the rectal Y cell.

### LIN-15A antagonizes the SynMuvB chromatin modifiers which act as barriers for Y-to-PDA

We next examined the role of SynMuvBs genes, a class of genes known to act redundantly with *lin-15A* to repress *lin-3/EGF* during VPC specification (reviewed in [51]). SynMuvBs code for members of chromatin complexes and modulators such as the DREAM, MEC or NuRD complexes (S1 Table) and are major negative regulators of gene expression [43,52–64].

We reasoned that in the Y cellular context, the SynMuvB genes could also act redundantly to LIN-15A. Under this hypothesis, we would expect single SynMuvB mutants to exhibit defects in Y-to-PDA Td, and/or double mutants *lin-15A + SynMuvB* to exhibit an increased defect penetrance (compared to the single *lin-15A* mutants). However, we found that no single SynMuvB mutant displayed defects in Y-to-PDA transdifferentiation. Moreover, the vast majority of the double mutants in combination with *lin-15A(n767)* actually displayed a defect suppression, from the typical 50% penetrance down to below 10% (Fig 4A-4C). Thus, absence of SynMuv B activities is not problematic for the Td to occur, as would be expected for activities blocking it. Such block becomes apparent in absence of LIN-15A, while LIN-15A requirement largely disappears when members of the SynMuvB factors are absent. These data suggest that SynMuvBs act as a brake for Y-to-PDA Td, and that one main function of LIN-15A is to antagonize it. Accordingly, we found that defects of *lin-56(n2728)* were also suppressed in double mutants with *lin-35(n745)*, *lin-15B(n744)* and *lin-13(ok838)* (S4A Fig).

**Fig 4:**
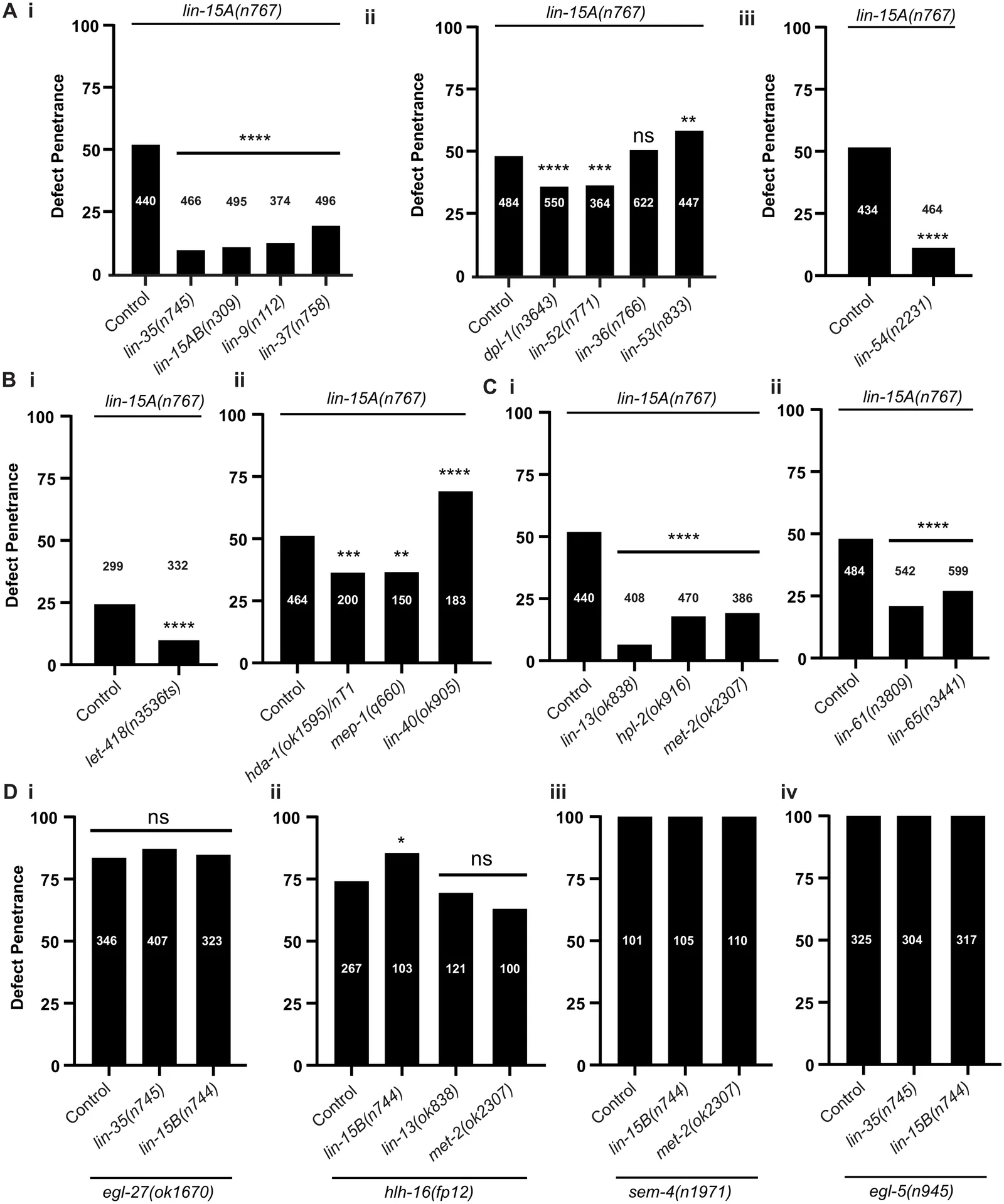
LIN-15A antagonizes the SynMuvB class of chromatin modifiers in Y-to-PDA. **A** Loss of members of the DREAM complex suppresses the Td defects of *lin-15A(n767)* mutants. X2 and Fisher’s exact tests with Bonferroni corrections for proportion comparisons. **B** Loss of the members of the NuRD and MEC complexes suppresses the Td defects of *lin-15A(n767)* mutants. *let-418(n3536)* is a temperature sensitive allele and was scored at 25°C. X2 and Fisher’s exact tests with Bonferroni corrections for proportion comparisons. **C** Loss of the SynMuvBs associated to the heterochromatin suppresses the Td defects of *lin-15A(n767)* mutants. X2 and Fisher’s exact tests with Bonferroni corrections for proportion comparisons. **D** Drivers are not modulated by SynMuvBs: Losses of SynMuvB chromatin modifiers do not suppress Td defects induced by the loss of the Driver factors EGL-27(**i**), HLH16 **(ii)**, SEM-4 (**iii**) or EGL-5 **(iv)**. X2 and Fisher’s exact tests with Bonferroni corrections for proportion comparisons.

Because MET-2 frequently acts together with other H3K9 methyltransferases, we also tested *set-25* and *set-32* mutants. In contrast to *met-2* loss, neither mutation modified the transdifferentiation defects of *lin-15A(n767)* (*set-25;lin-15A* mutant, n = 393; *set-32;lin-15A* mutant, n = 224), suggesting that the observed interaction is unlikely to reflect a generic requirement for H3K9 methyltransferases and instead points toward a more specific role of MET-2 within the SynMuvB identity-safeguarding machinery. As the function of the SynMuvBs and LIN-15A (as a SynMuvA) in VPC specification is to repress *lin-3/EGF*, and as the Y cell is rectal-epithelial, we also tested whether *lin-3/EGF* is involved in Td initiation. We assessed the effects of the loss-of-function alleles of *lin-3/EGF mt378* and *e1417*, and of the gain-of-function allele *n4441* [65], alone and in double mutants with *lin-15A(n767)*. In *n4441* a mutation in the promoter of *lin3/EGF* prevents its repression by the SynMuvAs including LIN-15A. We found that none of these *lin-3/EGF* alleles induced defects on their own, nor did they alter defect penetrance in *lin-15A(n767)* (S3 Fig). Hence, the functions of LIN-15A and of the SynMuvBs in Y-to-PDA are independent of *lin-3/EGF*.

### The SynMuvBs specifically interact with the Licensers LIN-15A and LIN-56

Next, we examined if the SynMuvBs were only antagonized by the LIN-15A and LIN-56, or if they also interacted with the Drivers (Fig 4D). We tested if loss of the SynMuvBs *lin-35*, *lin-15B*, *lin-13* or *met-2* suppressed Td defects in Driver mutants *egl-27(ok1670)*, *hlh-16(fp12)*, *sem-4(n1971)* and *egl-5(n945)*. No significant suppression was found in any combination (Fig 4D), confirming our model of two independent pathways (Fig 5). Together, SynMuvBs and LIN-15A form a parallel mechanism, acting antagonistically either in the same pathway or on the same targets, and the main function of LIN-15A lies in its interaction with the various SynMuvBs. Hence, we propose the term “Td Licensers” for factors that facilitate transdifferentiation by alleviating mechanisms that safeguard the initial identity. In contrast, Drivers actively promote cell-fate conversion independently and are indispensable for the transdifferentiation.

**Fig 5:**
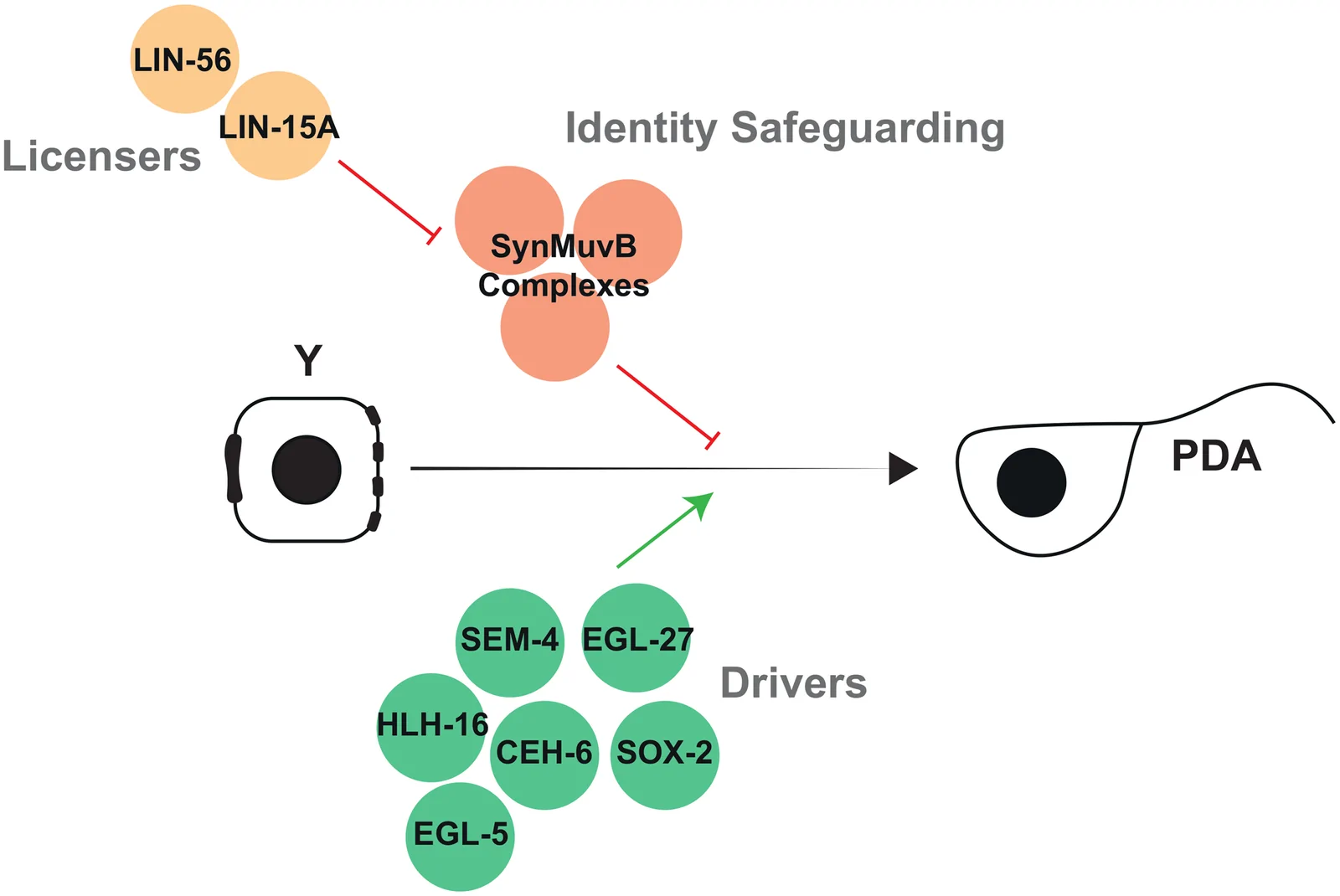
The Td Licensers LIN-15A and LIN-56 (yellow) antagonize chromatin-based mechanisms (red) that safeguard the initial rectal-epithelial identity. In parallel, Driver factors (green) promote Y-to-PDA Td initiation.

### The two NuRD complexes of *C. elegans* have opposite effects on Y plasticity

Out of all the SynMuvB genes tested, two stand out since their losses did not suppress the defects of *lin-15A(n767)* mutants (Fig 4A): loss of *lin-36* had no effect while loss *of lin-53* further significantly enhanced Td defects from 50% up to 60%. The absence of LIN-36 involvement in Y-to-PDA postmitotic Td [28] is consistent with its repression of genes associated with cell cycle progression [64].

However, loss of *lin-53* unambiguously increased the defects of *lin-15A* mutants. This is surprising, as LIN-53, HDA-1 and LET-418 are all components of the NuRD complex, yet loss of *hda-1* or *let-418* decreases the penetrance of *lin-15A(n767)* defects (Fig 4B). We found that the loss of another core component of the NuRD complex, *lin-40*, which is not a SynMuvB, also increased the defects of *lin-15A* mutants (Fig 4Bii). Two distinct NuRD complexes co-exist in *C. elegans* [66,67], which differ by their Mi2 homolog, either LET-418 (a SynMuvB) or CHD-3 (S5A Fig). We hypothesized that the opposite impact of loss of NuRD components on *lin-15A(0)* defects could be explained by different activities of the 2 NuRDs. Therefore, we specifically tested whether the two NuRD complexes could have opposite effects on cellular plasticity. Under this hypothesis, we predicted that if NuRD*LET*-418 restricts Y plasticity – as *let-418* loss suppresses *lin-15A(n767)* Td defects, then NuRD*CHD*-3 would further enhance it. As hypothesized, we found that the loss of *chd-3* increased Td defects in *lin-15A(n767)* mutants, similarly to the loss of *lin-40* or *lin-53*, while having no effect in WT backgrounds (S5B Fig). Thus, our data suggest the existence of two NuRD complexes with distinct roles in Y-to-PDA Td. These data are in accordance with previous reports of their distinct and sometimes opposite activities in other contexts [66,68].

Interestingly, CHD-3 loss also enhanced the defects observed in *egl-27(ok151)* mutants (S5C Fig). Since LIN-15A and EGL-27 act in two parallel pathways, these results suggest that the action of the NuRD*CHD*-3 complex is not specific to the Licensers/SynMuvBs nor the Drivers but act in a similar manner on both pathways (S5D Fig). Thus, Y-to-PDA involves a complex dance of various and close chromatin complexes that either facilitate it or block it.

### The SynMuvBs mainly act in the rectal cells for their role of barriers for Y-to-PDA transdifferentiation

To further address the role of the SynMuvBs in Y-to-PDA and their relationship with LIN-15A, we chose to focus on LIN-35, the sole *C. elegans* ortholog of pRb and core component of the SynMuvB-containing DREAM complex. We reasoned that since most DREAM component mutants display a similar genetic interaction with *lin-15A*, and have similar binding patterns [64], it is likely that they overall represent one machinery opposing Y-to-PDA initiation.

We first sought to identify in which tissue the *lin-35* was required for its role in Y-to-PDA initiation. Hence, we performed tissue-specific rescue experiments of LIN-35/DREAM in *lin-35(n745); lin-15A(n767)* animals (S4B Fig). We found that rescuing the expression of *lin-35* ubiquitously (under *eft-3p* promoter) or in the rectal area (under *egl-5p* promoter) consistently restored Td defects (S4B Fig). In contrast, intestinal (under *ugt-22p* or *elt-2p* promoters) and neuronal (under *rab-3p* promoter) expression led to much more limited and inconsistent rescues (S4B Fig). Given these results, we concluded that LIN-35/DREAM mainly acts in the rectal cells during Y-to-PDA. However, it may have another role in non-rectal tissues that contribute – to a much lesser degree – to its function as a brake for Td.

### LIN-15A does not affect SynMuvB expression in Y

We next aimed to understand how LIN-15A antagonizes LIN-35/DREAM and MET-2 in Y-to-PDA transdifferentiation. The first possibility is that LIN-15A represses their expression, or their nuclear localization. We compared the expression and the localization of endogenously tagged LIN-35 and MET-2 in WT animals and in *lin-15A(n767)* mutants. We observed that both proteins were expressed and strictly nuclear in *lin-15A(n767)*, as previously described for WT [64,69]. Because we showed that LIN-35/DREAM was crucial in the rectal cells for its role in Td, we measured the fluorescence intensity within the nucleus of the Y cell. We found that the expression of LIN-35/DREAM was not altered by the loss of *lin-15A* (S4C Fig) and no obvious change was detected in any of the other rectal cells either. We observed that the MET-2 signal was slightly lower in *lin-15A(n767)* mutants compared to the WT (S4D Fig), while the opposite would be expected if LIN-15A repressed *met-2* expression. Hence, the antagonization of the SynMuvBs by LIN-15A is not likely mediated by a negative control of their expression but rather by impacting their activity.

### LIN-15A modulates the binding of the DREAM complex

To better understand the functional antagonism between the DREAM complex and LIN-15A, which both bind chromatin [56,64] (S6 Fig), we sought to compare their bound regions and dependencies through Chromatin Immuno-Precipitation (ChIP)-seq experiments. While the DREAM complex represses cycle genes in combination with THAP domain protein LIN-36, it represses germline genes in the soma in combination with THAP domain protein LIN-15B [64]. Given that LIN-15A possesses a THAP domain required for its role in Y-to-PDA (Fig 1D), that involves no cell division, and binds onto naked and nucleosome-bound DNA (S6A and S6B Fig), we reasoned that LIN-15A also acts on chromatin, and might modulate DREAM activity. To investigate whether the genetic antagonism between LIN-15A and SynMuvB factors could be associated with changes in chromatin occupancy, we performed ChIP-seq in synchronized unstarved L1 larvae. Directly testing this possibility during Y-to-PDA transdifferentiation was not technically feasible, as sufficient material could not be obtained from the single Y cell and the experiments required tightly synchronized fed animals. We therefore analyzed whole-animal populations using antibodies against endogenous LIN-35/DREAM, LIN-15B and LIN-15A in WT, *lin-15A(fp31)* (CRISPR knock-out of the entire *lin-15A* locus), *lin-15B(n744)* (null) and *lin-35(n745)* (null) mutants, reasoning that organism-wide changes in chromatin occupancy could reveal a mechanism consistent with the genetic interactions observed during transdifferentiation.

We found 4926 and 6330 peaks bound by LIN-35/DREAM and LIN-15B respectively, with 87,0% shared genes (n = 3676) in WT animals (S2 Table). LIN-35/DREAM and LIN-15B had the majority of their peaks (61,4% for LIN-35 and 54,3% for LIN-15B) located in accessible promoter regions (S6C Fig) while binding patterns are consistent with previous data obtained from L1 larvae synchronized through starvation (95% and 89,9% overlap for LIN-35/DREAM and LIN-15B respectively with [64]; S2 Table). LIN-15A was found to bind 5260 regions (S2 Table), the majority of which are also located in coding promoter regions (58,7%, S6C Fig), and overlap (n = 2937) (89,4%) with LIN-35/DREAM and/or LIN-15B (S6D Fig). For all three proteins, the promoter-bound genes are enriched for similar GO terms related to metabolic and developmental pathways (e.g., TOR, IIS or Wnt, S2 Table).

It was previously shown that LIN-15B and LIN-35 exhibit co-regulation and dynamic binding at shared targets [64]. Given the extensive overlap between LIN-15A, LIN-35 and LIN-15B binding profiles, we asked whether the genetic antagonism observed between LIN-15A and SynMuvB factors could be associated with changes in chromatin occupancy. As previously reported in starved L1 larvae [64], hundreds of LIN-35 and LIN-15B peaks displayed reduced or increased signal when the other factor was absent (Fig 6A and S2 Table). Similarly, increased and decreased LIN-15A peaks were observed in *lin-35* and *lin-15B* mutants (Fig 6A and S2 Table). In contrast, very few LIN-35 or LIN-15B peaks showed reduced occupancy in *lin-15A* mutants, whereas approximately 100 peaks exhibited increased signal in the absence of LIN-15A (Figs 6A and S6E and S2 Table). Thus, loss of LIN-15A is associated with increased LIN-35 and LIN-15B occupancy at a subset of loci. This organism-wide pattern is coherent with the genetic antagonism observed between LIN-15A and SynMuvB factors during Y-to-PDA transdifferentiation. While we found no significant GO enrichment for the small sets of genes associated with increased LIN-35 (n = 85) or LIN-15B (n = 105) signal, these observations raise the possibility that modulation of SynMuvB chromatin occupancy contributes to the antagonistic relationship between LIN-15A and SynMuvB factors (Fig 6B).

**Fig 6:**
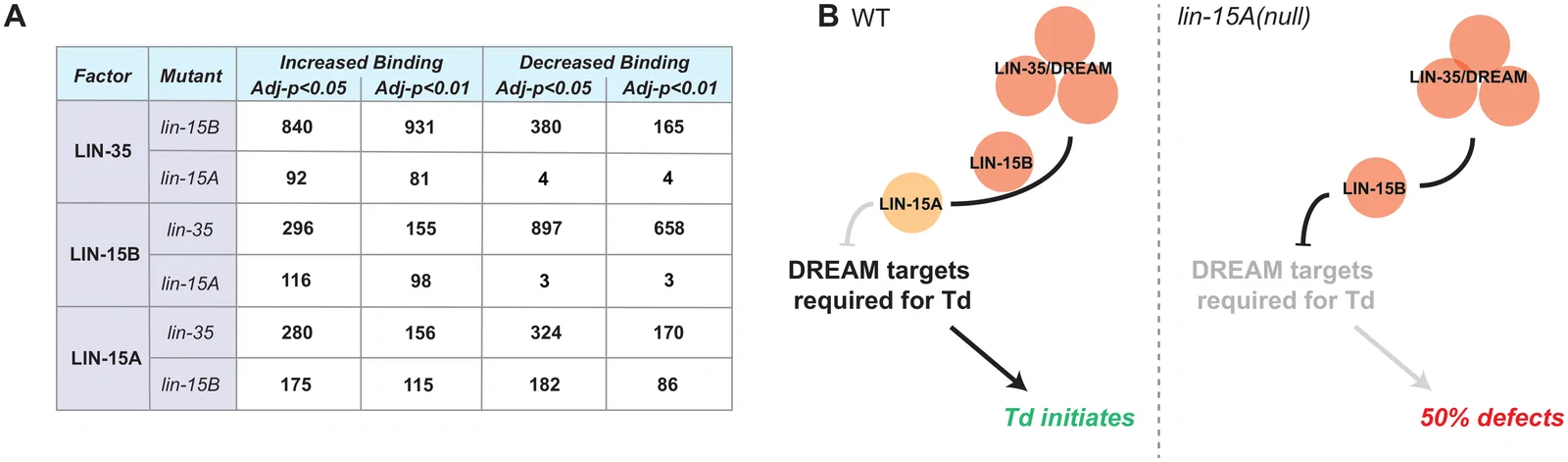
LIN-15A binds chromatin and can modulate DREAM binding onto a subset of its targets. **A** Number of peaks differentially bound by LIN-35/DREAM, LIN-15B and LIN-15A in the different mutant backgrounds. Loss of *lin-15A* increases the binding of LIN-35/DREAM (92 peaks corresponding to 85 genes) and LIN-15B (116 peaks corresponding to 105 genes) much more than it decreases it (4 and 3 peaks respectively), suggesting that LIN-15A only prevents DREAM complex binding, on a subset of its target genes. In contrast, losses of *lin-15B* or *lin-35* have mixed effects on LIN-35/DREAM or LIN-15B binding. Similarly, the losses of *lin-35* or *lin-15B* have mixed effects on LIN-15A binding. **B** Proposed molecular mechanism of LIN-15A, LIN-35/DREAM and LIN-15B interaction in Y-to-PDA. LIN-15A may shield certain DREAM targets relevant for Td from DREAM gene repression activity. In *lin-15A* mutants (right), those targets are not protected from DREAM-mediated repression, and get turned off, which impairs Td initiation.

## Discussion

### Drivers and licensers

Specific differentiated cells maintain the ability to change their identity even at later stages of development, in a process called natural transdifferentiation [21,33,70]. In *C. elegans*, the rectal Y cell, that transdifferentiates into the PDA motoneuron, is a well-characterized example [28,33,34]. We previously found that several factors, together designated as ‘plasticity cassette’, are required for the initiation step [36,37]. In this study, we have found that a second pathway centered on the SynMuvA LIN-15A is also required for Y-to-PDA. LIN-15A, a nuclear factor able to bind chromatin, acts in a cooperative manner with LIN-56, to enable Td initiation. Importantly, LIN-15A and LIN-56 act in parallel to the previously described factors. We further found that the Td defects in *lin-15A* mutants, but not in plasticity cassette mutants, could be suppressed by the loss of most SynMuvBs (Figs 4 and S4). Altogether, our genetic data argue for LIN-15A, LIN-56 and SynMuvBs to act antagonistically to each other and in parallel to plasticity cassette factors. Hence, we propose a two-pathway model (Fig 5): on the one hand, conserved plasticity factors (NODE-like complex) are absolutely required to dedifferentiate the rectal Y cell, acting as ‘Drivers’. Their losses result in 100% (or close to 100%) defects in initiation. On the other hand, LIN-15A and LIN-56 are required to facilitate the initiation of Y-to-PDA (their loss does not induce full Td defect penetrance), through antagonization of SynMuvB activity, therefore acting as Td ‘Licensers’. Importantly, these Td Licensers do not represent a cell cycle-coupled mechanism, as they are required for Tds occurring in absence (Y-to-PDA in hermaphrodite) or presence (Y-to-PDA in males) of cell division. This work further shows that even for a differentiated cell that is developmentally programmed to transdifferentiate, reprogramming barriers still exist, that are powered by several chromatin factors and complexes. Thus, the SynMuvB brake on Y-to-PDA natural Td parallel the need to remove chromatin modifiers to enable experimentally-induced reprogramming both in *C. elegans* [71–74] and in other systems (reviewed in [6,75]). These results raise several questions. To what extent do different transdifferentiation events rely on common identity-safeguarding mechanisms? Conversely, are Licensers themselves conserved across transdifferentiation events, or context-dependent?

### Context specificity of the Td licensers

Our data suggest that LIN-15A and LIN-56 Td Licenser activity is context-dependent. LIN-15A and LIN-56 are required for Y-to-PDA Td in both hermaphrodites and males, but not in K-to-DVB Td, although it occurs in the same organ, shortly before Y-to-PDA, also requires several members of the NODE-like complex and K expresses LIN-15A [32]. It is possible that not all differentiated cells benefit from identity safeguarding mechanisms. Alternatively, other Td Licensers may act in different Td contexts. While the control of chromatin remodeling and TF activity are key recurring themes, different cellular identities could be maintained by distinct safeguarding mechanisms, requiring different barriers-alleviating mechanisms before reprogramming can occur. Consistent with this idea, studies from the Hobert and Tursun groups have highlighted the diversity of factors that restrict induced reprogramming [71–74,76].

Furthermore, during embryonic development LIN-15A can even restrict cellular plasticity (Fig 3E). Hence its role to promote plasticity appears specific to certain cellular identities, and this is paralleled with the changing nature of its interactions with SynMuvBs: acting redundantly with SynMuvB factors to repress vulval development or embryonic multipotency, while promoting plasticity during Y-to-PDA by antagonizing SynMuvB activity. Likewise, LIN-53 has been shown to block experimentally-induced reprogramming of germ cells [71], while our data indicate that it promotes natural Y-to-PDA Td alongside LIN-15A. This suggests that the same chromatin factors can either restrict or facilitate plasticity, and that the nature of the interactions between these chromatin modifiers and the precise cocktail safeguarding a given differentiated identity is likely context-specific. Chromatin factors diversity may provide for modular flexibility where different combinations would have different functional outcomes. Our finding that the two *C. elegans* NuRD complexes exert opposite effects on Y-to-PDA further illustrates the functional flexibility of chromatin regulatory machineries.

### The SynMuvBs as identity-safeguarding barriers to transdifferentiation

The SynMuvBs, which likely act in the Y cell, do not appear to impact on the Y cell identity itself: Y is formed in all examined SynMuvB mutants and the expression of rectal markers is unaffected by the loss of *lin-15A* (S1C Fig). Hence, their activity likely only provides robust maintenance of the differentiated Y identity once established.

SynMuvBs have been described as chromatin factors, the majority of which are part of different multimeric complexes, and act to enforce an inactive chromatin state. We suggest that these chromatin factors repress in Y transcriptional programs associated with alternative cellular identities, thereby reinforcing the differentiated rectal identity of the Y cell. De-repression of one or more alternative transcriptomes would allow progression towards an alternative fate, here neuronal. Such role would be consistent with their role in other cellular contexts [52–54,56–60,62–64]. For instance, SynMuvBs antagonize germline fate in the intestine [55] and NuRD is required for maintaining muscle integrity [77]. In this framework, Td Licensers would facilitate transdifferentiation by alleviating identity-safeguarding chromatin states, thereby creating a permissive context for the Drivers to execute the Td program. On the other hand, the Drivers role would be to activate Td genes or inactivate rectal genes. Another non-exclusive hypothesis is that SynMuvBs could repress the genes that make the Y cell plastic and able to initiate the Td process.

### The SynMuvBs may act as a single machinery in Y-to-PDA

We found that LIN-15A antagonizes all of the SynMuvBs, except LIN-53 because of its role in the two alternative NuRD complexes (Figs 4A and 4B and S5). This suggests that the SynMuvBs act as a single multimodal machinery in Y-to-PDA. Supporting this idea, work from the Ahringer lab has highlighted that the binding regions of LET-418/Mi2, HPL-2, LIN-61, LIN-13 and MET-2 - all involved in antagonizing Y-to-PDA - extensively overlap [78], and that LIN-35/DREAM and LIN-15B repress their targets via H3K9me2 deposition by MET-2 [64]. Notably, *set-25* and *set-32* mutants did not phenocopy *met-2* loss, supporting a specific role for MET-2 within the SynMuvB machinery in Y-to-PDA.

Similar interactions between SynMuvB complexes have also been reported in mammalian cells, where Sin3B, a protein associated with HDAC (orthologue of NuRD component HDA-1) interacts with the DREAM complex and is required for repression of its targets to promote cell quiescence [79]. Hence, LIN-15A may antagonize a single machinery involved in gene repression. Supporting this view of regulation at the chromatin level, LIN-15A THAP domain – for DNA binding – is required for its role in Y-to-PDA (Figs 1D and S6). Such central Licensers that antagonize several chromatin complexes acting as one barrier could provide leads to improve the efficiency of experimentally-induced reprogramming strategies.

### The antagonism between LIN-15A and the SynMuvBs may take place at the chromatin level

Since the SynMuvBs may act as a single machinery in Y-to-PDA, we focused on the possible interaction between LIN-15A and the DREAM complex. Previous studies highlighted that the latter acts through THAP domain proteins, notably LIN-15B [56,64]. Since LIN-15A is in an operon with LIN-15B, also possesses a THAP domain, required for its role in Td, and can bind DNA (Figs 1D and S6), we reasoned that it could also interact with LIN-35/DREAM, and point to a potential mechanistic explanation for the genetic antagonism observed between LIN-15A and SynMuvB factors. We found extensive overlap between the binding regions of LIN-35/DREAM, LIN-15B and LIN-15A (Fig 6A and 6B). These results are consistent with previous records of LIN-35/DREAM and LIN-15B binding [64]. As LIN-35/DREAM and LIN-15B repress gene expression via MET-2 H3K9*me*2 promoter marking, these findings raise the possibility that LIN-15A influences SynMuvB chromatin occupancy and associated transcriptional regulation.

In accordance with this hypothesis, loss of LIN-15A was associated with increased LIN-35/DREAM and LIN-15B occupancy at a subset of loci. Although these experiments were performed on whole-animal populations and therefore cannot directly address chromatin regulation during Y-to-PDA transdifferentiation, they provide a plausible mechanistic explanation for the genetic antagonism observed between LIN-15A and SynMuvB factors. Given the established role of SynMuvB proteins in MET-2-dependent promoter marking, one possibility is that LIN-15A facilitates transdifferentiation by alleviating identity-safeguarding chromatin states at a subset of targets.

### Antagonization of pRb in the context of reprogramming

LIN-35 is the sole pocket protein encoded in the *C. elegans* genome. Its mammalian counterpart, the retinoblastoma protein (pRb), is a major tumor suppressor, whose loss plays a critical role in the development and growth of multiple cancers. Most of the functions of pocket proteins - notably cell cycle control and differentiation - are conserved throughout eukaryotes, including vertebrates, nematodes, and even plants [80,81]. Importantly, one of the major anti-oncogenic roles of mammalian pRb is to inhibit cancer cell EMT [82]. Thus, it is possible that *C. elegans* LIN-35 retained a similar ability to oppose cell-fate plasticity in differentiated cells. pRb has also been shown to repress the reprogramming of murine fibroblast into iPSCs [83]. Y-to-PDA transdifferentiation appears to represent another, conserved role of pocket proteins in regulating reprogramming. Kareta and colleagues equally showed that this function of pRb was independent on its role in regulating the cell cycle, data reflecting our own report that LIN-36 - the DREAM adaptor mediating LIN-35 repression on cell cycle-associated genes [64], does not play any role in Y Td. Altogether, these findings suggest that pocket proteins may have a conserved ability to restrict cellular plasticity, independently of their control of the cell cycle.

### Limitations of this study

As no Y-specific driver is currently available, we cannot directly test if LIN-15A and/or LIN-35/DREAM specifically act in the Y cell, among the rectal cells where the *egl-5p* promoter is active. Nevertheless, the cell-autonomous functions of these factors in gene regulation, together with the genetic and rescue data presented here, are consistent with a model in which LIN-15A acts within the Y cell. The ChIP-seq analyses performed in this study could only be carried out at the whole animal level and therefore cannot directly identify the chromatin changes occurring during Y-to-PDA transdifferentiation. Consequently, Y-specific targets relevant to plasticity may have been missed. This limitation comes from technical reasons. The small size - approximately 2 *μ*m – of the epithelial-rectal Y cell wrapped in the resistant L1 cuticle and the requirement for tightly synchronized fed L1 animals currently prevents the collection of sufficient material (*>*3000 individual cells) for cell-specific chromatin profiling. Although the chromatin data suggest a possible mechanism underlying the genetic antagonism between LIN-15A and SynMuvB factors, they do not establish causality between occupancy changes and transdifferentiation outcomes. For ChIP-seq experiments, we focused on the interaction between LIN-15A and the DREAM complex based on our genetic data (Fig 4), and on reports that the DREAM complex acts notably through LIN-15B, in an operon with LIN-15A and sharing similar THAP domains [64]. We reasoned that SynMuvBs act as a single machinery in Y-to-PDA, but we cannot exclude that LIN-15A and other SynMuvBs interact independently of LIN-35/DREAM.

## Materials and methods

### *C. elegans* culture, Y-to-PDA scorings and general experimental procedures

*Caenorhabditis elegans* strains (S3 Table) were maintained according to standard protocols [84] - 20°C, OP50 on Nematode Growth Media (NGM) agar plates. For all experiments, a parental population was amplified. Broad synchronization was performed by hypochlorite treatment (bleach), embryos were next placed on fresh plates (max 3 days old), seeded with fresh bacterial lawns (1 day old O/N liquid culture) dried after seeding at 37°C for a few hours. OP50 loans were grown from single colony to saturation in Luria-Bertani Broth (LB) at 37°C, shaking at 170rpm. This procedure was found critical to have consistent penetrance of the *lin-15A* mutants. Presence or absence of the PDA motor neuron (scoring) were performed at the late L3, L4 or young adult stage. Defects were defined by the absence of PDA (no cell body, no axon, no marker expressed). PDA presence was assessed with the integrated array *syIs63 (cog-1p::GFP)* or *wyIs75 (exp-1p::GFP)*. Defect penetrance does not depend on the marker used for mutants involved in the initiation step. K-to-DVB was scored in a similar fashion, using the integrated array *wyIs75 (unc-47p::RFP)*. Experiments were conducted in triplicates at minima. Except stated otherwise, all experiments were conducted at strictly 20°C, with OP50 as a food source on NGM plates. Scoring of *let-418(n3536)* was notably conducted differently. *n3536* is a temperature sensitive allele of *let-418* behaving as a null at 25°C (C. Wicky *pers. comm*). At 25°C, *n3536* leads to a developmental arrest. In order to use *n3536* but leave time for Y-to-PDA to proceed, without being impacted by the developmental arrest, synchronized newly-hatched larvae were placed at 20°C and transferred at 25°C 6 hours later. This procedure allows *let-418(n3536)* to develop reaching the L3 and L4 stages. The efficiency of this procedure was confirmed by the induction of the Multivulva phenotype in *lin-15A(n767); let-418(n3536ts)* mutants, the irreversible developmental arrest and subsequently the reduced penetrance of Y-to-PDA defects. Of note, the Td defect penetrance of *lin-15A(n767)* mutants is decreased at 25°C compared 20°C from 50% down to 25%.

For epifluorescence microscopy, animals were immobilized on 2% agarose pads using Tricaine

0.4% and Tetramisole 0.04% in UP water. Leica DM6 B microscope was used, and images were captured with LAS X software through Hamamatsu Digital Camera C11440. Standard image analyses were performed with FIJI.

*C. elegans* transgenic strains were engineered by DNA microinjection into the gonad cytoplasm of young adults [85]. The mix of injected DNA was always composed by the plasmid of interest together with a co-injection marker and pBSK for a final concentration of DNA of 200–250 ng/*μ*l in water or injection buffer (2% polyethylene glycol MW 6000–8000, 20 mM potassium phosphate pH 7.5, 3 mM potassium citrate pH 7.5). Transgenics F1 were cloned and the transmission of the plasmid array was evaluated in the F2 generation. Lines with a good transmission rate were usually kept. All the other strains used for this study have been obtained by crossing existing strains from our lab, other labs (obtained through CGC or directly) or from SunyBiotech for KI reporter strains which have been designed in the lab. The presence of mutations was always confirmed prior to the experiments by PCR genotyping for deletions and by PCR plus restriction digestion genotyping for point mutations [86]. Lysates of entire worms were produced for genotyping. Worms were lysed in a solution containing ultrapure water, PCR buffer 1X and proteinase K 0.5 mM, for 1 h at 65°C and then at 95°C for 15 min.

### Statistical analyses

Statistical analyses were performed with PRISM GraphPad 10. For all experiments, the statistical tests used are indicated in the corresponding figure legend. ns: p > 0.05, *: p < 0.05, **: p < 0.01, ***: p < 0.001, ****: p < 0.0001.

### Isolation and cloning of *fp22*

*fp22* was isolated from an EMS screen as a “no PDA” mutant by absence of the expected staining by *syIs63* transgenic marker. The mutant was backcrossed and EMS variant deep sequence mapping was performed as described in [42].

### RNA interference

For *lin-8*, *lin-38*, *smo-1* and *uba-2* (S3 Table), a gene fragment from a cDNA library made from RNA extracted from N2 worms was PCR amplified with primers containing flanking T7 promoter sequences. *In vitro* transcription using the PCR products as templates and T7 RNA polymerase was performed, and single stranded RNA was purified using Qiagen RNeasy Mini Kit (74104) and allowed to anneal as dsRNA by gradually lowering the temperature of the sample. dsRNA was injected into *rrf-3(pk1426); syIs63* or *rrf-3(pk1426); syIs63; lin-15A(n767)* adults and F1 progeny derived from these adults were scored for Y-to-PDA defects.

#### Tissue specific, truncated domains and heat-shock inducible rescues of LIN-15A and LIN-35.

Coding sequence were cloned from a complementary DNA (cDNA) library prepared from WT (N2) RNA that had been extracted using RNA-Solv. Promoter sequences were cloned from WT (N2) DNA. All final constructs were sequenced verified before use.

#### *lin-15A* rescue constructslin-15A rescue constructs.

The *lin-15p::mcherry::lin-15A cDNA* transgenic plasmid pSJ805 contains the *lin-15* promoter derived from N2 DNA via PCR using the primers F: 5’- GGTCGACCGGCCGACCGTTTTCTTTCG-3’ and R: 5’- GGGGATCCAATTACCTGAAAATATAAAAAAAATAATAAATTA-

TTCGGTCATCATGC-3’ and cloned in between the Acc1 and MscI sites of pPD49.78 (Addgene plasmid 1447; http://n2t.net/-addgene:1447; RRID:Addgene 1447).

*mcherry* sequence was amplified from pSJ9016 [17] using the primers F: 5’-

CCCTTGGAGGGTACC-CAGCTG-3’ and R: 5’-CATTCCTCCCTTATACAATTCATCCATGCCACC-3’ and cloned into the NheI site of the subsequent plasmid.

*lin-15A cDNA* was amplified from genomic N2 DNA via PCR using the primers F5’- ATGTTG-

GCTCCAGCGGCTCCAGCTAAAGATGTTGTCTCG-3’ and R 5’- CGCTTAAAAAATTGGCTCAGGCTTTGGATCC-3’. *lin-15A* cDNA was cloned by Gibson assembly using the primers F 5’-GGGAGGAATG-TTGGCTCCAGCGGCTCC-3’ and R 5’-CTGCGGCCGCTTAAAAAATTGGCTCAGGCTTTGG-3’.

For the *egl-5(6.5kb)p::lin-15A cDNA::SL2::mcherry* transgenic plasmid *lin-15A* cDNA was amplified from pSJ805 using the primers F: 5’-AAAAGATCTTATCTGACTCCGTGCATAC-3’ and R: 3’-AGGCCTTTATGTCCGGTGGCATCTC-5’and cloned into the Acc65I restriction site of pSJ962 Zuryn et al. [17].

For the *egl-5(6.5kb)p::lin-15A cDNA dCterm::SL2::mcherry* transgenic plasmid *lin-15A* cDNA lacking the C-term was amplified from pSJ805 using the primers F: 5’-CTACGCCAGATCTGCTAGCGCG-3’ and R: 3’-AGGCCTTTATGTCCGGTGGCATCTC-5’ and cloned into the Acc65I restriction site of pSJ962.

For the *egl-5(6.5kb)p::lin-15A cDNA dNterm::SL2::mcherry* transgenic plasmid *lin-15A* cDNA lacking the N-term was amplified from pSJ805 using the primers F: 5’-AAAAGATCTTATCTGACTCCGTGCATAC-3’ and R: 3’-GAATTCATGCATAGGCCTGCG-5’ and cloned into the Acc65I restriction site of pSJ962.

For the *egl-5(6.5kb)p::lin-15A cDNA dZnF::SL2::mcherry* transgenic plasmid point mutations were induced at C212S and C215S using mutagenesis PCR with the primers F: 5’CCTATCTGACTCCGGGCATACTCTGC -3’ and R: 3’- GCAGAGTATGCCCGGAGTCAGATAGG -5’ in pSJ805. The mutated *lin-15A* cDNA was amplified using the primers F:

5’-AAAAGATCTTATCTG-ACTCCGTGCATAC-3’ and R: 3’-AGGCCTTTATGTCCGGTGGCATCTC-5’ and cloned into the Acc65I restriction site of pSJ962.

The *hsp-16.2p::lin-15A cDNA::mCherry* transgenic plasmid pSJ830 was generated by cloning the *mcherry::lin-15A* cDNA sequence in between the BstBI and PvuI sites of pSJ9016 Zuryn et al. [17] into the AvaI site of pSJ805. *mcherry::lin-15A* cDNA was amplified from pSJ805 using the following primers 5’- CCGGGATTGGCCAAAGGACCCAAAGGTATGTTTCGAATG-3’ and 5’- CGGTCCTCCG-ATCGTTGTCAGAAGTAAGTTGGC-3’.

#### *lin-35* tissue-specific rescue constructslin-35 tissue-specific rescue constructs.

For the *egl-5(1.3kb)p::lin-35 cDNA::mkate* transgenic plasmid *pSJ835 lin-35* cDNA was cloned by Gibson assembly into pSJ834 in between the *delta-pes-10* and *mkate* sequences using the primers F: 5’-ATGCCGAAACGAGCAGCCGATG-3’ and R: 5’-ATCTCCAGACCGTTCCATAGCG-3’.

For the *ugt-22p::lin-35 cDNA::mkate* transgenic plasmid 830 bp of *ugt-22* promoter sequence was cloned into pSJ835 restriction sites HindIII and XbaI using the primers F: 5’AGCTTGCATGCC-TGCAGGTCGAC-3’ and R: 5’-CCGGTACCCTCCAAGGGTCCTC-3’.

For the *eft-3p::lin-35 cDNA::mkate* transgenic plasmid 610 bp of *eft-3* promoter sequence was cloned into pSJ835 restriction sites HindIII and XbaI using the primers F: 5’- ATGAAATA-

AGCTTGCATGCCTGCAGGCCTTGGTCGACGCACCTTTGGTCTTTTATTG-3’ and R: 5’-GGATCCTCTAGAGAGCAAAGTGTTTCCCAACT-3’.

For the *rab-3p::lin-35 cDNA::mkate* transgenic plasmid 1211 bp of *rab-3* promoter sequence was cloned into pSJ835 restriction sites HindIII and XbaI using the primers F:5’ ATGGATACGCTAACAACTTGGAAATGAAATAAGCTTATCTTCAGATGGGAGCAGTGGAC-3’ and R: 5’-CTACAGTAGCCCTATTTTCAGATGATCTCTAGAGGATCCCCGG-

GGATTGGCCAAAGGACC–3”

For the *elt-2p::lin-35 cDNA::mkate* transgenic plasmid 2896 bp of *elt-2* promoter sequence was cloned into pSJ835 restriction sites HindIII and XbaI using the primers F: 5’

GAAATAAGCTTTTGCATGCCTTCGTGTGATGG - 3’ and R: 5’- GGATCCTCTAGA-GATCTATAATCTATTTTCTAG – 3”

### Immunofluorescence

Whole-mount immunofluorescence was performed on synchronized L3 worms using a slightly modified Finney-Ruvkun procedure [34]. Primary antibody mouse anti-EGL-5 (1/100) was a gift from Scott Emmons and rabbit anti-AJM-1 (1/400) was obtained from Developmental Studies Hybridoma Bank. Secondary staining was performed using either Cy3-conjugated (Jackson ImmunoResearch Laboratories) or Alexa 488-conjugated (Invitrogen) antibodies. Imaging was performed using a Yokogawa CSU-W1 Leica DMi8 spinning disk microscope.

### Western blots

Whole worm protein extracts (mixed embryos and L1 larvae) prepared using RETSCH Mixer Mill MM 400 were resuspended into the double distilled water and mixed with appropriate volume of 4X-SDS loading buffer (Total- 50–100 *μ*l). Samples were heated at 95°C for 5 mins on a PCR machine followed by a quick centrifugation before loading on an SDS-PAGE. The SDS-PAGE (10–12%) was used to separate proteins. Samples were then transferred onto a PVDF membrane using standard wet western blot transfer methods. Blot transfer was achieved by running blot set-up at 120V for 120 at 4°C. This condition was sufficient for transfer for all molecular weight protein. After transfer, the membrane was blocked with TBST buffer with 5% skimmed milk with gentle shaking using rockers for 1hr at RT. The membrane was incubated with the appropriate primary antibody – Anti-LIN-15A Q2015 (1:1000 dilution in TBST buffer with 2.5% skimmed milk) and kept with gentle shaking using rockers for overnight at 4°C. The next day, the membrane was washed thrice at RT for 10 minutes in TBST buffer then incubated with HRP conjugated Goat anti-mouse secondary antibodies (GAM, made in-house (0.5 *μ*l in 10 ml of TBST (1/20000 dilution) and 5 ml used for one blot)) for 120 mins at RT on mild shaking. Blots were washed thrice for 10 minutes in TBST buffer and developed by Thermo Scientific™ SuperSignal™ West Pico PLUS Chemiluminescent Substrate (Catalog number 34577) as manufacturer’s protocol.

### LIN-15A streptavidine and FLAG pull-downs

For both experiments, 1 ug of LIN15A-FLAG tagged purified protein was incubated with 400 ng of Biotinylated DNA or 400ng of Biotinylated Recombinant or 400ng of Biotinylated Native Mononucleosomes (from HeLa cells) in 100mM NaCl. Samples were pull down with either Streptavidine-agarose bead or FLAG-agarose beads resuspended in SB2X, loaded on a 8%-12% SDS PAGE gradient gels and revealed with anti-FLAG or anti-H3 antibodies. For the FLAG pull-down, half of the pulled-down sample was spotted on a nylon membrane, UV crosslinked and revealed with an anti-streptavidine-HRP conjugated antibody.

### Generation of *lin-15A(fp31)* and *lin-15A(syb978)* alleles

Constructs containing Cas9 and sgRNAs with PAM sites before *lin-15A* start codon and after *lin-15* TATA box were injected together with Co-CRISPR sgRNA (pML3441/3442 against *dpy-10*) and repair template (OCG250 *rol-6*), as well as co-injection marker pCF390 into *lin-65(n3411); syIs63*. In F1 worms were selected for [Dpy]/[Rol] and co-injection marker, in F2 worms were selected for [Muv] phenotype and validated by sequencing. *lin-65(n3411)*, Co-CRISPR constructs and co-injection marker were crossed out or negatively selected to obtain *lin-15A(fp31); syIs63*.

The following constructs containing the sgRNAs were used:

- pSJ407 sgRNA 1.6 *lin-15A* start
- PSJ408 sgRNA 1.9 *lin-15A* start
- pSJ409 sgRNA 2.6 *lin-15A* start
- pSJ410 sgRNA 2.8 *lin-15A* start
- pSJ411 sgRNA 3.7 *lin-15A* 3’UTR
- pSJ412 sgRNA 3.8 *lin-15A* 3’UTR
- pSJ413 sgRNA 4.8 *lin-15A* stop
- pSJ414 sgRNA 4.8 *lin-15A* stop

Generation of endogenously tagged LIN-15A::mCherry *syb978* was outsourced to Suny Biotech. The *mCherry* sequence was added in Nter, right after *lin-15A* ATG, and separated by the ‘6xHis’ plus ‘linker’ sequence CATCACCATCACCATCACGGAGGAAACGGAAA-CAACGCTGCCGGA

### Cell fate challenge assays

Blastomere multipotency was assessed through the overexpression of the muscle-determining transcription factor HLH-1 under a heat-shock inducible promoter following Fukushige and Krause’s [49] procedure. Strains carried an integrated array containing *hlh-1* cDNA under the control of the *hsp-16.41* promoter [87] and a *rol-6* phenotypic marker as well as a muscle-specific fluorescent reporter *ccIs4251*, inducing the expression of the GFP in the nucleus and the mitochondria of muscle-determined blastomeres. The precise protocol for this assay can be found in (Lambert and Jarriault, *in prep*).

### Chromatin-immuno precipitation sequencing (ChIP-seq) of LIN-15A, LIN-15B and LIN-35/DREAM

For each genotype, four to five independent synchronized L1 fed populations were generated and processed through chromatin preparation. Because synchronization of fed L1 larvae without starvation results in limited amount of starting material, not all preparations passed the quality control criteria required for downstream analysis. Final analyses were performed using the subset of high-quality biological replicates available for each condition, resulting in a minimum of two biological replicates per experiment.

Synchronization of the L1 larvae was obtained through series of hatch-pulses. Briefly, WT, *lin-15A(fp31)*, *lin-15B(n744)* and *lin-35(n745)* animals were grown on peptone plates with *E. coli* HB101 as a food source (http://www.wormbook.org/chapters/wwwtrans-formationmicroinjection/transformationmicroinjection.htmld0e878) and then bleached. Eggs were deposited on empty NGM plates which were washed with M9 every three hours. Collected animals were immediately frozen into liquid nitrogen (‘pearls’). Samples from the successive washes were pulled together. *fp31* is a deletion of the *lin-15A* loci (*lin-15B* unchanged). *n744* and *n745* are premature stop codons, respectively in LIN-15B and LIN-35. Worm pearls were ground into a powder using a RETSCH Mixer Mill MM 400 (13 seconds at 25 Htz) and subsequently pre-incubated in PBS. Fixation was performed by addition of 1.5 mM EGS (Pierce 21565) for 4 minutes, followed by the addition of formaldehyde to a final concentration of 1%, and incubated for a further 4 minutes. Fixation was stopped by the addition of 0.125 M glycine for 5 minutes. Fixed tissues were washed 2X with PBS with protease inhibitors (Roche EDTA-free protease inhibitor cocktail tablets 05056489001) and once in FA buffer (50 mM HEPES pH7.5, 1 mM EDTA, 1% Triton X-100, 0.1% sodium deoxycholate, and 150 mM NaCl) with protease inhibitors (FA+), then resuspended in 1 mL FA+ buffer per 1 mL of ground worm powder. The extracts were sonicated to an average size of 250 base pairs using a Bioruptor Pico (Diagenode), and 15 *μ*g of DNA was used per ChIP reaction. The following antibodies (all from the Ahringer lab) were used for the Chromatin-Immunoprecipitation: Q2015 – anti-LIN-15A (this study), Q2330 – anti-LIN-15B [62], Q2001 – anti-LIN-35 [64]. ChIP-seq and library preparations were performed as described in [88].

Genome and canonical gene annotations were downloaded from Wormbase (release 285), retaining only protein coding, pseudogenes and lincRNAs. ChIP-seq data were preprocessed using trim galore (available at https://github.com/FelixKrueger/TrimGalore, version 0.6.7) and mapped using bwa mem [89] (version 0.7.17). No exogenous spike-in chromatin or DNA controls were included. RPGC-normalized (reads per genomic content) coverage tracks from mapq10 reads were generated using bamCoverage from the deepTools2 suite [90](version 3.5.5). Peaks from each replicate were called using MACS2 [91] (version 2.2.9.1) using no control sample and the following parameters: –call-summits –bdg –SPMR –gsize ce –keep-dup all –nomodel. To define a set of peaks for each wild-type or mutant condition, MACS2 summits with a score >6 were first extended to 100 bp on each side. Peaks from each replicate condition were then intersected using the intersect function from BED-tools [92] (version 2.30.0), and the intersected peaks retained only if the MACS2 score from each replicate was between 0.1- and 10-fold of the score from the other replicate (or the average of the other replicates, when three replicates were available). This was done to mitigate the impact of between-replicate variation in the quantification of differentially bound regions (see below). Intersected peaks were then rescaled to 200 bp around their midpoint. PCA analysis and correlation plots among wild-type and mutant replicates from each factor were carried out using the deepTools2 suite.

Sites showing differential enrichment of each factor between the wild-type and each mutant were defined using the R (version 4.2.0) package DESeq2 [93] (version 1.38.0). For this analysis, all peaks for each factor called in the wild type and mutant conditions were combined using the merge function from BEDtools, and the ChIP-seq data were quantified in each peak using summarizeOverlaps (mode = ”Union”). Annotation of the promoters were performed according to [94] for comparison with the LIN-15B and LIN-35 peaks in the WT backgrounds of starved L1s from [64], and using wormbase WBCel235 annotation to identify the closest predicted gene (intragenic, or upstream peaks ≤2kb from the ATG).

Gene Ontology (GO) and Kyoto Encyclopedia of Genes and Genomes (KEGG) pathway enrichment analyses were carried out with ShinyGO 0.85 [95–97], on peaks with an adjusted Pvalue<0,05, using *C. elegans* genome as background. Pathways & GOs were selected based on FDR and sorted by their respective Fold Enrichment. The Groups tool from ShinyGo allowed to retrieve annotations for the genes corresponding to up-bound peaks for LIN-35 and LIN-15B in *lin-15A* mutants.

## Supporting information

S1 Fig *lin-15A* and *lin-56* are required for Y-to-PDA transdifferentiation. **A** Allele complementation shows that *fp22* and *n767* are recessive, null alleles of *lin-15A*. **B** *lin-15A* and *lin-56* are the only SynMuvAs involved in Y-to-PDA transdifferentiation. Inactivation via mutant alleles or RNA interference of the other SynMuvAs does not induce any Y-to-PDA defect on their own, nor enhance the Y-to-PDA defective phenotypes of *lin-15A(n767)* mutant. X2 and Fisher’s exact tests with Bonferroni corrections were used for proportion comparisons. **C** A normal Y rectal cell is formed in *lin-15A* mutant: Expression of rectal-epithelial markers *col-34* and *lin-26* is not affected by loss of *lin-15A* prior to Td initiation. Representative pictures of the rectal area during the L1 stage. The rectal slit is indicated by a red bar. Scalebars = 5 *μ*m. **D** The transgenes used in LIN-15A rescue-experiments are well expressed in Y prior to Td initiation. Images of the rectal area in L1 animals, the Y cell is indicated. Scalebars = 5 *μ*m. **E** LIN-15A is nuclear and widely expressed, including in the Y cell (indicated) from mid-embryonic development to late L1 stage. LIN-15A is expressed in Y during the L1 stage, prior to Td. Red bar: rectal slit. Scalebars embryo = 20 *μ*m. Scalebars L1 larvae = 10 *μ*m. In all pictures depicting the rectal area, Anterior is to the left and ventral to the bottom.
(TIF)

S2 Fig LIN-15A does not regulate CEH-6 nor SOX-2 expression. **A** CEH-6 expression is not affected by the loss of *lin-15A* (WT, n = 45 / *lin-15A(n767)*, n = 50). **B** SOX-2 expression is not affected by the loss of *lin-15A* (WT, n = 48 / *lin-15A(n767)*, n = 54). The Y cell is indicated. L1 worms were imaged. Red bar: rectal slit.
(TIF)

S3 Fig LIN-15A function in Y-to-PDA is independent of LIN-3. **A** Loss of function (*mt378* and *e1417*) and gain of function (*n4441*) alleles of *lin-3* do not induce defects in Y-to-PDA transdifferentiation. The total number of animals scored is indicated above each genotype. **B** LIN-15A function in Y-to-PDA is independent of *lin-3*. The penetrance of *lin-15A(n767)* is not affected by loss or gain of function mutations of *lin-3*. X2 and Fisher exact tests for proportion comparisons; ns, not statistically significant. The total number of animals scored is indicated above each genotype.
(TIF)

S4 Fig SynMuvBs likely act in the Y cell and are not regulated by *lin-15A.* **A** Loss of the SynMuvBs LIN-15B, LIN-35 or LIN-13 suppresses the defects of *lin-56(n2728)* mutants. The total number of animals scored is indicated above each genotype. X2 and Fisher exact tests with Bonferroni corrections for proportion comparisons: ns: p > 0.05, *: p < 0.05, **: p < 0.01, ***: p < 0.001, ****: p < 0.0001. **B i** Tissue-specific rescue of LIN-35/pRb. Ubiquitous and rectal-specific rescues of LIN-35/pRb strongly anti-suppress Td defects in double *lin-35(n745); lin-15A(n767)* mutant. Intestinal and neuronal rescues have weaker and/or inconsistent effect. **ii** Expression of the different constructs used in the *lin-35* rescue experiments (quantified in B i) in the rectal area of L1 animals (prior to Td initiation). Scalebar = 10 *μ*m. **C i** Quantification of the expression levels of endogenously-tagged LIN-35/pRb in the Y cell in L1 animals (prior to Td). Levels are unchanged in *lin-15A(n767)* compared to control animals suggesting either post-translational or functional regulation. Shapiro–Wilk test for normality. Student t-test for mean comparisons. **C ii** Representative images of *lin-35* expression in the rectal area of L1 animals using Nomarski and fluorescence optics. Red: the rectal marker EGL-5::mCherry CRISPR allele. Green: the endogenous LIN-35::GFP CRISPR allele. The Y cell is indicated as well as P12.pa which replaces Y in the rectum after Td. P12.pa does not express EGL-5::mCherry. Yellowbar: rectal slit. Scalebar = 10 *μ*m. **D i** Quantification of the expression levels of endogenously-tagged MET-2 in the Y cell in L1 animals (prior to Td). Levels are slightly decreased in *lin-15A(n767)* compared to control animals demonstrating that LIN-15A does not repress MET-2 expression. Student t-test for mean comparisons. **D ii** Representative images of *met-2* expression in the rectal area of L1 animals using Nomarski and fluorescence optics. Green: Y marker HLH-16::GFP CRISPR allele. Red: the endogenous MET-2::mCherry CRISPR allele. The Y cell is outlined in white, the rectal slit in yellow. Scalebar = 10 *μ*m.
(TIF)

S5 Fig The two NuRD complexes of *C. elegans* have distinct and opposite effects on the plasticity of Y. **A** Overview of the components of the two NuRD and of the MEC complexes and their respective influence on cellular plasticity. **B** Loss of the Mi2 homolog CHD-3, specific to one of the two NuRDs, enhances the Td defects in *lin-15A(n767)* mutants but has no effect in the WT background. The *chd-3(eh4)* allele, which contains a 2kb deletion that removes most of the CHD-3 helicase domain, is likely null [66]. Fisher exact test for proportion comparisons. The total number of animals scored is indicated above each genotype. **C** Loss of the CHD-3 also enhances the Td defects in *egl-27(ok151)* mutants. Fisher exact test for proportion comparisons. **D** CHD-3 acts as an enhancer for cellular plasticity in the two parallel pathways.
(TIF)

S6 Fig LIN-15A binds chromatin, similarly to LIN-35 and LIN-15B. **A** Streptavidine pull-down experiments with 1*μ*g of LIN15A-FLAG tagged purified protein incubated with 400ng of Biotinylated DNA (DNA-Bio) or 400ng of Biotinylated Recombinant (Rec Mononucl-Bio) or 400ng of Biotinylated Native Mononucleosomes and pulled down with Streptavidine agarose beads. Pulldowns were loaded on a 8%- > 12% SDS PAGE gradient gel and revealed with anti-FLAG or anti-H3 antibodies. **B** FLAG pull down experiments with 1*μ*g of LIN15A-FLAG tagged purified protein incubated with 400ng of Biotinylated DNA (DNA-Bio) or 400ng of Biotinylated Recombinant (Rec Mononucl-Bio) or 400ng of Biotinylated Native Mononucleosomes and FLAG agarose beads. ½ of the pull-down was revealed with anti-FLAG or anti-H3 antibodies. ½ of the pull-down was revealed with an anti-streptavidine-HRP coniugated antibody. **C** LIN-15A, LIN-35 and LIN-15B binds similar regions. LIN-15A binds mainly onto gene promoters (58,7%), similarly to LIN-15B (54,3%) and LIN-35 (61,4%). A total of 4926 (LIN-35), 6330 (LIN-15B), 5260 (LIN-15A) peaks were bound (See S2 Table, sheets #2 and #22, #6 and #23, #10 and #24). Fraction of total peaks on different genomic elements as defined by Serizay et al. [94] and repetitive sequences as defined by McMurchy et al. [78], per factor. If a peak was assigned to two non-repetitive elements it is counted twice; the number of peaks in multiple elements is indicated below each pie chart. If a peak is counted for a non-repetitive element and a repetitive sequence it is only counted as non-repetitive elements. **D** Overlap of promoter-bound genes by LIN-15A, LIN-15B and LIN-35/pRb. All three factor overlap on a large fraction of their promoter-bound genes (n = 2937) corresponding to 77,5% of LIN-15A, 77,4% of LIN-35 and 70,7% of LIN-15B promoter bound-genes. **E** Representative examples of differential binding identified by ChIP-seq. Browser tracks showing increased LIN-35 (i) or LIN-15B (ii) occupancy in *lin-15A(fp31)* mutants relative to WT. Individual biological replicates are displayed using identical y-axis scaling within each panel. Gene annotations are shown below.
(TIF)

S1 TableList of the SynMuvB (SMB) and chromatin factors examined in this work.SMBs are chromatin-associated factors or complexes that negatively regulate gene expression. The associated chromatin complex, the mutant allele used, its impact on the penetrance of Y-to-PDA defect in *lin-15A(n767)* background, as well as the defect in the single *lin-15A(n767)* mutant scored in parallel (“control’ ’) are indicated. Significance: $\chi^{2}$ and Fisher’s exact tests.(TIF)

S2 Table Raw and analyzed ChIP-seq data for LIN-15A, LIN-15B and LIN-35/DREAM in wild-type and mutant backgrounds. (XLSX)

S3 Table List of the strains and RNAi clones used in this study. (XLSX)

S1 Data File containing all the original data (except for ChIP-seq data) supporting the findings of this study. (XLSX)

## References

[1] Gurdon JB. The developmental capacity of nuclei taken from differentiating endoderm cells of Xenopus laevis. J Embryol Exp Morphol. 1960;8:505–26. doi: 10.1242/dev.8.4.505 13709842

[2] Gurdon JB. The developmental capacity of nuclei taken from intestinal epithelium cells of feeding tadpoles. J Embryol Exp Morphol. 1962;10:622–40. doi: 10.1242/dev.10.4.622 13951335

[3] Takahashi K, Yamanaka S. A decade of transcription factor-mediated reprogramming to pluripotency. Nat Rev Mol Cell Biol. 2016;17(3):183–93. doi: 10.1038/nrm.2016.8 26883003

[4] Wang H, Yang Y, Liu J, Qian L. Direct cell reprogramming: approaches, mechanisms and progress. Nat Rev Mol Cell Biol. 2021;22(6):410–24. doi: 10.1038/s41580-021-00335-z 33619373 PMC8161510

[5] Zhang Y-X, Chen S-L, Li Y-M, Zheng Y-W. Limitations and challenges of direct cell reprogramming in vitro and in vivo. Histol Histopathol. 2022;37(8):723–37. doi: 10.14670/HH-18-458 35417038

[6] Rothman J, Jarriault S. Developmental plasticity and cellular reprogramming in Caenorhabditis elegans. Genetics. 2019;213(3):723–57. doi: 10.1534/genetics.119.302333 PMC6827377 31685551

[7] Yuan S, Norgard RJ, Stanger BZ. Cellular Plasticity in Cancer. Cancer Discovery. 2019;9(7):837–51. doi: 10.1158/2159-8290.cd-19-0015 PMC6606363 30992279

[8] Takahashi K, Yamanaka S. Induction of pluripotent stem cells from mouse embryonic and adult fibroblast cultures by defined factors. Cell. 2006;126(4):663–76. doi: 10.1016/j.cell.2006.07.024 16904174

[9] Huels DJ, Medema JP. Think About the Environment: Cellular Reprogramming by the Extracellular Matrix. Cell Stem Cell. 2018;22(1):7–9. doi: 10.1016/j.stem.2017.12.006 29304343

[10] Apostolou E, Hochedlinger K. Chromatin dynamics during cellular reprogramming. Nature. 2013;502(7472):462–71. doi: 10.1038/nature12749 24153299 PMC4216318

[11] Patel T, Hobert O. Coordinated control of terminal differentiation and restriction of cellular plasticity. eLife. 2017. doi: 10.7554/elife.24100 PMC5397285 28422646

[12] Mall M, Kareta MS, Chanda S, Ahlenius H, Perotti N, Zhou B, et al. Myt1l safeguards neuronal identity by actively repressing many non-neuronal fates. Nature. 2017;544(7649):245–9. doi: 10.1038/nature21722 28379941 PMC11348803

[13] Lee J, Taylor CA, Barnes KM, Shen A, Stewart EV, Chen A. A Myt1 family transcription factor defines neuronal fate by repressing non-neuronal genes. eLife. 2019. doi: 10.7554/elife.46703 PMC6684318 31386623

[14] Missinato MA, Murphy S, Lynott M, Yu MS, Kervadec A, Chang Y-L, et al. Conserved transcription factors promote cell fate stability and restrict reprogramming potential in differentiated cells. Nat Commun. 2023;14(1):1709. doi: 10.1038/s41467-023-37256-8 36973293 PMC10043290

[15] Özcan I, Tursun B. Identifying Molecular Roadblocks for Transcription Factor-Induced Cellular Reprogramming In Vivo by Using C. elegans as a Model Organism. J Dev Biol. 2023;11(3):37. doi: 10.3390/jdb11030037 37754839 PMC10531806

[16] Blau HM, Baltimore D. Differentiation requires continuous regulation. J Cell Biol. 1991;112(5):781–3. doi: 10.1083/jcb.112.5.781 1999456 PMC2288865

[17] Zuryn S, Ahier A, Portoso M, White ER, Morin M-C, Margueron R, et al. Transdifferentiation. Sequential histone-modifying activities determine the robustness of transdifferentiation. Science. 2014;345(6198):826–9. doi: 10.1126/science.1255885 25124442

[18] Sousounis K, Qi F, Yadav MC, Millán JL, Toyama F, Chiba C, et al. A robust transcriptional program in newts undergoing multiple events of lens regeneration throughout their lifespan. Elife. 2015;4:e09594. doi: 10.7554/eLife.09594 26523389 PMC4739772

[19] Eguchi G, Abe SI, Watanabe K. Differentiation of lens-like structures from newt iris epithelial cells in vitro. Proc Natl Acad Sci U S A. 1974;71(12):5052–6. doi: 10.1073/pnas.71.12.5052 4216028 PMC434038

[20] Selman K, Kafatos FC. Transdifferentiation in the labial gland of silk moths: is DNA required for cellular metamorphosis? Cell Differ. 1974;3(2):81–94. doi: 10.1016/0045-6039(74)90030-x 4277742

[21] Okada TS. Transdifferentiation: Flexibility in Cell Differentiation. Oxford: Clarendon Press; 1991.

[22] Henry JJ, Tsonis PA. Molecular and cellular aspects of amphibian lens regeneration. Prog Retin Eye Res. 2010;29(6):543–55. doi: 10.1016/j.preteyeres.2010.07.002 20638484 PMC3463140

[23] Kodama R, Eguchi G. From lens regeneration in the newt to in-vitro transdifferentiation of vertebrate pigmented epithelial cells. Semin Cell Biol. 1995;6(3):143–9. doi: 10.1006/scel.1995.0020 7548853

[24] Yamada T. Control mechanisms in cell-type conversion in newt lens regeneration. vol. 13 of Monographs in Developmental Biology. Basel: S. Karger; 1977. 197401

[25] Schmid V, Reber-Müller S. Transdifferentiation of isolated striated muscle of jellyfish in vitro: the initiation process. Semin Cell Biol. 1995;6(3):109–16. doi: 10.1006/scel.1995.0016 7548849

[26] Merrell AJ, Stanger BZ. Adult cell plasticity in vivo: de-differentiation and transdifferentiation are back in style. Nat Rev Mol Cell Biol. 2016;17(7):413–25. doi: 10.1038/nrm.2016.24 26979497 PMC5818993

[27] Sulston JE, Horvitz HR. Post-embryonic cell lineages of the nematode, Caenorhabditis elegans. Dev Biol. 1977;56(1):110–56. doi: 10.1016/0012-1606(77)90158-0 838129

[28] Jarriault S, Schwab Y, Greenwald I. A Caenorhabditis elegans model for epithelial-neuronal transdifferentiation. Proc Natl Acad Sci U S A. 2008;105(10):3790–5. doi: 10.1073/pnas.0712159105 18308937 PMC2268801

[29] Riddle MR, Weintraub A, Nguyen KCQ, Hall DH, Rothman JH. Transdifferentiation and remodeling of post-embryonic C. elegans cells by a single transcription factor. Development. 2013;140(24):4844–9. doi: 10.1242/dev.103010 24257624 PMC3848185

[30] Sammut M, Cook SJ, Nguyen KCQ, Felton T, Hall DH, Emmons SW, et al. Glia-derived neurons are required for sex-specific learning in C. elegans. Nature. 2015;526(7573):385–90. doi: 10.1038/nature15700 26469050 PMC4650210

[31] Molina-García L, Lloret-Fernández C, Cook SJ, Kim B, Bonnington RC, Sammut M. Direct glia-to-neuron transdifferentiation gives rise to a pair of male-specific neurons that ensure nimble male mating. eLife. 2020. doi: 10.7554/elife.48361 PMC7609048 33138916

[32] Riva C, Hajduskova M, Gally C, Suman SK, Ahier A, Jarriault S. A natural transdifferentiation event involving mitosis is empowered by integrating signaling inputs with conserved plasticity factors. Cell Rep. 2022;40(12):111365. doi: 10.1016/j.celrep.2022.111365 36130499 PMC9513805

[33] Lambert J, Lloret-Fernández C, Laplane L, Poole RJ, Jarriault S. On the origins and conceptual frameworks of natural plasticity-Lessons from single-cell models in C. elegans. Curr Top Dev Biol. 2021;144:111–59. doi: 10.1016/bs.ctdb.2021.03.004 33992151

[34] Richard JP, Zuryn S, Fischer N, Pavet V, Vaucamps N, Jarriault S. Direct in vivo cellular reprogramming involves transition through discrete, non-pluripotent steps. Development. 2011;138(8):1483–92. doi: 10.1242/dev.063115 21389048 PMC3188571

[35] White JG, Southgate E, Thomson JN, Brenner S. The structure of the nervous system of the nematode Caenorhabditis elegans. Philos Trans R Soc Lond B Biol Sci. 1986;314(1165):1–340. doi: 10.1098/rstb.1986.0056 22462104

[36] Kagias K, Ahier A, Fischer N, Jarriault S. Members of the NODE (Nanog and Oct4-associated deacetylase) complex and SOX-2 promote the initiation of a natural cellular reprogramming event in vivo. Proc Natl Acad Sci U S A. 2012;109(17):6596–601. doi: 10.1073/pnas.1117031109 22493276 PMC3340080

[37] Daniele T, Cury J, Morin M-C, Ahier A, Isaia D, Jarriault S. Essential and dual effects of Notch activity on a natural transdifferentiation event. Nat Commun. 2025;16(1):75. doi: 10.1038/s41467-024-55286-8 39746948 PMC11697417

[38] Rashid A, Tevlin M, Lu Y, Shaham S. A developmental pathway for epithelial-to-motoneuron transformation in C. elegans. Cell Rep. 2022;40(13):111414. doi: 10.1016/j.celrep.2022.111414 PMC9579992 36170838

[39] Clark SG, Lu X, Horvitz HR. The Caenorhabditis elegans locus lin-15, a negative regulator of a tyrosine kinase signaling pathway, encodes two different proteins. Genetics. 1994;137(4):987–97. doi: 10.1093/genetics/137.4.987 7982579 PMC1206075

[40] Huang LS, Tzou P, Sternberg PW. The lin-15 locus encodes two negative regulators of Caenorhabditis elegans vulval development. Mol Biol Cell. 1994;5(4):395–411. doi: 10.1091/mbc.5.4.395 8054684 PMC301050

[41] Davison EM, Saffer AM, Huang LS, DeModena J, Sternberg PW, Horvitz HR. The LIN-15A and LIN-56 Transcriptional Regulators Interact to Negatively Regulate EGF/Ras Signaling inCaenorhabditis elegansVulval Cell-Fate Determination. Genetics. 2011;187(3):803–15. doi: 10.1534/genetics.110.124487 PMC3063674 21196525

[42] Zuryn S, Le Gras S, Jamet K, Jarriault S. A strategy for direct mapping and identification of mutations by whole-genome sequencing. Genetics. 2010;186(1):427–30. doi: 10.1534/genetics.110.119230 20610404 PMC2940307

[43] Fay DS, Yochem J. The SynMuv genes of Caenorhabditis elegans in vulval development and beyond. Dev Biol. 2007;306(1):1–9. doi: 10.1016/j.ydbio.2007.03.016 17434473 PMC1955466

[44] Sulston JE, Schierenberg E, White JG, Thomson JN. The embryonic cell lineage of the nematode Caenorhabditis elegans. Dev Biol. 1983;100(1):64–119. doi: 10.1016/0012-1606(83)90201-4 6684600

[45] Mahoney TR, Luo S, Round EK, Brauner M, Gottschalk A, Thomas JH, et al. Intestinal signaling to GABAergic neurons regulates a rhythmic behavior in Caenorhabditis elegans. Proc Natl Acad Sci U S A. 2008;105(42):16350–5. doi: 10.1073/pnas.0803617105 18852466 PMC2570992

[46] Wang H, Girskis K, Janssen T, Chan JP, Dasgupta K, Knowles JA, et al. Neuropeptide secreted from a pacemaker activates neurons to control a rhythmic behavior. Curr Biol. 2013;23(9):746–54. doi: 10.1016/j.cub.2013.03.049 23583549 PMC3651789

[47] Beg AA, Ernstrom GG, Nix P, Davis MW, Jorgensen EM. Protons act as a transmitter for muscle contraction in C. elegans. Cell. 2008;132(1):149–60. doi: 10.1016/j.cell.2007.10.058 18191228 PMC2258244

[48] Coraggio F, Püschel R, Marti A, Meister P. Polycomb and Notch signaling regulate cell proliferation potential during Caenorhabditis elegans life cycle. Life Sci Alliance. 2019;2(1):e201800170. doi: 10.26508/lsa.201800170 PMC6306570 30599047

[49] Fukushige T, Krause M. The myogenic potency of HLH-1 reveals wide-spread developmental plasticity in early C. elegans embryos. Development. 2005;132(8):1795–805. doi: 10.1242/dev.01774 15772130

[50] Yuzyuk T, Fakhouri THI, Kiefer J, Mango SE. The polycomb complex protein mes-2/E(z) promotes the transition from developmental plasticity to differentiation in C. elegans embryos. Dev Cell. 2009;16(5):699–710. doi: 10.1016/j.devcel.2009.03.008 19460346 PMC2693235

[51] Shin H, Reiner DJ. The Signaling Network Controlling C. elegans Vulval Cell Fate Patterning. J Dev Biol. 2018;6(4):30. doi: 10.3390/jdb6040030 30544993 PMC6316802

[52] Fay DS, Keenan S, Han M. fzr-1 and lin-35/Rb function redundantly to control cell proliferation in C. elegans as revealed by a nonbiased synthetic screen. Genes Dev. 2002;16(4):503–17. doi: 10.1101/gad.952302 11850412 PMC155341

[53] Unhavaithaya Y, Shin TH, Miliaras N, Lee J, Oyama T, Mello CC. MEP-1 and a homolog of the NURD complex component Mi-2 act together to maintain germline-soma distinctions in C. elegans. Cell. 2002;111(7):991–1002. doi: 10.1016/s0092-8674(02)01202-3 12507426

[54] Wang D, Kennedy S, Conte D Jr, Kim JK, Gabel HW, Kamath RS, et al. Somatic misexpression of germline P granules and enhanced RNA interference in retinoblastoma pathway mutants. Nature. 2005;436(7050):593–7. doi: 10.1038/nature04010 16049496

[55] Petrella LN, Wang W, Spike CA, Rechtsteiner A, Reinke V, Strome S. synMuv B proteins antagonize germline fate in the intestine and ensure C. elegans survival. Development. 2011;138(6):1069–79. doi: 10.1242/dev.059501 21343362 PMC3042865

[56] Latorre I, Chesney MA, Garrigues JM, Stempor P, Appert A, Francesconi M, et al. The DREAM complex promotes gene body H2A.Z for target repression. Genes Dev. 2015;29(5):495–500. doi: 10.1101/gad.255810.114 25737279 PMC4358402

[57] Wu X, Shi Z, Cui M, Han M, Ruvkun G. Repression of germline RNAi pathways in somatic cells by retinoblastoma pathway chromatin complexes. PLoS Genet. 2012;8(3):e1002542. doi: 10.1371/journal.pgen.1002542 22412383 PMC3297578

[58] Käser-Pébernard S, Müller F, Wicky C. LET-418/Mi2 and SPR-5/LSD1 cooperatively prevent somatic reprogramming of C. elegans germline stem cells. Stem Cell Rep. 2014;2(4):547–59. doi: 10.1016/j.stemcr.2014.02.007 24749077 PMC3986580

[59] Goetsch PD, Garrigues JM, Strome S. Loss of the Caenorhabditis elegans pocket protein LIN-35 reveals MuvB’s innate function as the repressor of DREAM target genes. PLoS Genet. 2017;13(11):e1007088. doi: 10.1371/journal.pgen.1007088 29091720 PMC5683655

[60] Ahringer J, Gasser SM. Repressive chromatin in Caenorhabditis elegans: establishment, composition, and function. Genetics. 2018;208(2):491–511. doi: 10.1534/genetics.117.300386 PMC5788517 29378810

[61] Costello ME, Petrella LN. C. elegans synMuv B proteins regulate spatial and temporal chromatin compaction during development. Development. 2019;146(19):dev174383. doi: 10.1242/dev.174383 31515206 PMC6803374

[62] Rechtsteiner A, Costello ME, Egelhofer TA, Garrigues JM, Strome S, Petrella LN. Repression of Germline Genes in Caenorhabditis elegans Somatic Tissues by H3K9 Dimethylation of Their Promoters. Genetics. 2019;212(1):125–40. doi: 10.1534/genetics.118.301878 30910798 PMC6499516

[63] Kipreos ET, van den Heuvel S. Developmental Control of the Cell Cycle: Insights from Caenorhabditis elegans. Genetics. 2019;211(3):797–829. doi: 10.1534/genetics.118.301643 30846544 PMC6404260

[64] Gal C, Carelli FN, Appert A, Cerrato C, Huang N, Dong Y, et al. DREAM represses distinct targets by cooperating with different THAP domain proteins. Cell Rep. 2021;37(3):109835. doi: 10.1016/j.celrep.2021.109835 34686342 PMC8552245

[65] Saffer AM, Kim DH, van Oudenaarden A, Horvitz HR. The Caenorhabditis elegans Synthetic Multivulva Genes Prevent Ras Pathway Activation by Tightly Repressing Global Ectopic Expression of lin-3 EGF. PLoS Genetics. 2011;7(12):e1002418. doi: 10.1371/journal.pgen.1002418 PMC3248470 22242000

[66] Käser-Pébernard S, Pfefferli C, Aschinger C, Wicky C. Fine-tuning of chromatin composition and Polycomb recruitment by two Mi2 homologues during C. elegans early embryonic development. Epigenet Chromat. 2016;9(1). doi: 10.1186/s13072-016-0091-3 PMC5024519 27651832

[67] von Zelewsky T, Palladino F, Brunschwig K, Tobler H, Hajnal A, Müller F. The C. elegans Mi-2 chromatin-remodelling proteins function in vulval cell fate determination. Development. 2000;127(24):5277–84. doi: 10.1242/dev.127.24.5277 11076750

[68] Passannante M, Marti C-O, Pfefferli C, Moroni PS, Kaeser-Pebernard S, Puoti A, et al. Different Mi-2 complexes for various developmental functions in Caenorhabditis elegans. PLoS One. 2010;5(10):e13681. doi: 10.1371/journal.pone.0013681 21060680 PMC2965115

[69] Delaney CE, Methot SP, Guidi M, Katic I, Gasser SM, Padeken J. Heterochromatic foci and transcriptional repression by an unstructured MET-2/SETDB1 co-factor LIN-65. J Cell Biol. 2019;218(3):820–38. doi: 10.1083/jcb.201811038 30737265 PMC6400574

[70] Eguchi G, Kodama R. Transdifferentiation. Curr Opin Cell Biol. 1993;5(6):1023–8. doi: 10.1016/0955-0674(93)90087-7 8129939

[71] Tursun B, Patel T, Kratsios P, Hobert O. Direct conversion of C. elegans germ cells into specific neuron types. Science. 2011;331(6015):304–8. doi: 10.1126/science.1199082 21148348 PMC3250927

[72] Patel T, Tursun B, Rahe DP, Hobert O. Removal of Polycomb repressive complex 2 makes C. elegans germ cells susceptible to direct conversion into specific somatic cell types. Cell Rep. 2012;2(5):1178–86. doi: 10.1016/j.celrep.2012.09.020 23103163 PMC3529301

[73] Hajduskova M, Baytek G, Kolundzic E, Gosdschan A, Kazmierczak M, Ofenbauer A, et al. MRG-1/MRG15 Is a Barrier for Germ Cell to Neuron Reprogramming in Caenorhabditis elegans. Genetics. 2019;211(1):121–39. doi: 10.1534/genetics.118.301674 30425042 PMC6325694

[74] Kolundzic E, Ofenbauer A, Bulut SI, Uyar B, Baytek G, Sommermeier A, et al. FACT Sets a Barrier for Cell Fate Reprogramming in Caenorhabditis elegans and Human Cells. Dev Cell. 2018;46(5):611-626.e12. doi: 10.1016/j.devcel.2018.07.006 30078731 PMC6137076

[75] Brumbaugh J, Di Stefano B, Hochedlinger K. Reprogramming: identifying the mechanisms that safeguard cell identity. Development. 2019;146(23). doi: 10.1242/dev.182170 PMC6918747 31792064

[76] Rahe DP, Hobert O. Restriction of Cellular Plasticity of Differentiated Cells Mediated by Chromatin Modifiers, Transcription Factors and Protein Kinases. G3 Genes Genomes Genetics. 2019;9(7):2287–302. doi: 10.1534/g3.119.400328 PMC6643894 31088904

[77] Müthel S, Uyar B, He M, Krause A, Vitrinel B, Bulut S, et al. The conserved histone chaperone LIN-53 is required for normal lifespan and maintenance of muscle integrity in Caenorhabditis elegans. Aging Cell. 2019;18(6):e13012. doi: 10.1111/acel.13012 31397537 PMC6826145

[78] McMurchy AN, Stempor P, Gaarenstroom T, Wysolmerski B, Dong Y, Aussianikava D. A team of heterochromatin factors collaborates with small RNA pathways to combat repetitive elements and germline stress. eLife. 2017;6. doi: 10.7554/elife.21666 PMC5395297 28294943

[79] Bainor AJ, Saini S, Calderon A, Casado-Polanco R, Giner-Ramirez B, Moncada C, et al. The HDAC-Associated Sin3B Protein Represses DREAM Complex Targets and Cooperates with APC/C to Promote Quiescence. Cell Rep. 2018;25(10):2797–807.e8. doi: 10.1016/j.celrep.2018.11.024 30517867 PMC6324198

[80] Zluhan-Martínez E, Pérez-Koldenkova V, Ponce-Castañeda MV, Sánchez M de la P, García-Ponce B, Miguel-Hernández S, et al. Beyond What Your Retina Can See: Similarities of Retinoblastoma Function between Plants and Animals, from Developmental Processes to Epigenetic Regulation. Int J Mol Sci. 2020;21(14):4925. doi: 10.3390/ijms21144925 32664691 PMC7404004

[81] Desvoyes B, Gutierrez C. Roles of plant retinoblastoma protein: cell cycle and beyond. EMBO J. 2020;39(19):e105802. doi: 10.15252/embj.2020105802 32865261 PMC7527812

[82] Egger JV, Lane MV, Antonucci LA, Dedi B, Krucher NA. Dephosphorylation of the Retinoblastoma protein (Rb) inhibits cancer cell EMT via Zeb. Cancer Biol Ther. 2016;17(11):1197–205. doi: 10.1080/15384047.2016.1235668 27645778 PMC5137485

[83] Kareta MS, Gorges LL, Hafeez S, Benayoun BA, Marro S, Zmoos A-F, et al. Inhibition of pluripotency networks by the Rb tumor suppressor restricts reprogramming and tumorigenesis. Cell Stem Cell. 2015;16(1):39–50. doi: 10.1016/j.stem.2014.10.019 25467916 PMC4389904

[84] Brenner S. The genetics of Caenorhabditis elegans. Genetics. 1974;77(1):71–94. doi: 10.1093/genetics/77.1.71 PMC1213120 4366476

[85] Mello CC, Kramer JM, Stinchcomb D, Ambros V. Efficient gene transfer in C.elegans: extrachromosomal maintenance and integration of transforming sequences. EMBO J. 1991;10(12):3959–70. doi: 10.1002/j.1460-2075.1991.tb04966.x 1935914 PMC453137

[86] Morin M-C, Hoff-Yoessle S, Jarriault S. A simple PCR-based method to follow and genotype alleles with single nucleotide changes. MicroPubl Biol. 2020. doi: 10.17912/micropub.biology.000218 32550496 PMC7252334

[87] Stringham EG, Dixon DK, Jones D, Candido EP. Temporal and spatial expression patterns of the small heat shock (hsp16) genes in transgenic Caenorhabditis elegans. Mol Biol Cell. 1992;3(2):221–33. doi: 10.1091/mbc.3.2.221 1550963 PMC275521

[88] Jänes J, Dong Y, Schoof M, Serizay J, Appert A, Cerrato C. Chromatin accessibility dynamics across C. elegans development and ageing. eLife. 2018;7. doi: 10.7554/elife.37344 PMC6231769 30362940

[89] Li H, Durbin R. Fast and accurate short read alignment with Burrows-Wheeler transform. Bioinformatics. 2009;25(14):1754–60. doi: 10.1093/bioinformatics/btp324 19451168 PMC2705234

[90] Ramírez F, Ryan DP, Grüning B, Bhardwaj V, Kilpert F, Richter AS, et al. deepTools2: a next generation web server for deep-sequencing data analysis. Nucleic Acids Res. 2016;44(W1):W160-5. doi: 10.1093/nar/gkw257 27079975 PMC4987876

[91] Zhang Y, Liu T, Meyer CA, Eeckhoute J, Johnson DS, Bernstein BE, et al. Model-based analysis of ChIP-Seq (MACS). Genome Biol. 2008;9(9):R137. doi: 10.1186/gb-2008-9-9-r137 18798982 PMC2592715

[92] Quinlan AR, Hall IM. BEDTools: a flexible suite of utilities for comparing genomic features. Bioinformatics. 2010;26(6):841–2. doi: 10.1093/bioinformatics/btq033 20110278 PMC2832824

[93] Love MI, Huber W, Anders S. Moderated estimation of fold change and dispersion for RNA-seq data with DESeq2. Genome Biol. 2014;15(12):550. doi: 10.1186/s13059-014-0550-8 25516281 PMC4302049

[94] Serizay J, Dong Y, Jänes J, Chesney M, Cerrato C, Ahringer J. Distinctive regulatory architectures of germline-active and somatic genes in C. elegans. Genome Res. 2020;30(12):1752–65. doi: 10.1101/gr.265934.120 33093068 PMC7706728

[95] Ge SX, Jung D, Yao R. ShinyGO: a graphical gene-set enrichment tool for animals and plants. Bioinformatics. 2020;36(8):2628–9. doi: 10.1093/bioinformatics/btz931 31882993 PMC7178415

[96] Luo W, Brouwer C. Pathview: an R/Bioconductor package for pathway-based data integration and visualization. Bioinformatics. 2013;29(14):1830–1. doi: 10.1093/bioinformatics/btt285 23740750 PMC3702256

[97] Kanehisa M, Furumichi M, Sato Y, Ishiguro-Watanabe M, Tanabe M. KEGG: integrating viruses and cellular organisms. Nucleic Acids Res. 2021;49(D1):D545–51. doi: 10.1093/nar/gkaa970 33125081 PMC7779016

