# Supplementary material for: The SynMuvA lin-15A licenses natural transdifferentiation by antagonizing identity safeguarding mechanisms
Source: PLoS Genet. 2026 Sep 25;22(9):e1012290. doi: 10.1371/journal.pgen.1012290 (PMC13641710; doi:10.1371/journal.pgen.1012290)
Supplement: Supplementary file 1 [file pgen.1012290.s011.pdf]

Full Revision

Manuscript number: #RC-2026-03533
Corresponding author(s): Sophie, Jarriault

[Please use this template only if the submitted manuscript should be considered by the affiliate journal as a full revision in response to the points raised by the reviewers.

If you wish to submit a preliminary revision with a revision plan, please use our "Revision Plan" template. ***It is important to use the appropriate template to clearly inform the editors of your intentions.****]*

1. General Statements [optional]

This section is optional. Insert here any general statements you wish to make about the goal of the study or about the reviews.

We thank the reviewers for their insightful comments. We believe that the broader significance of this work lies in the identification of endogenous mechanisms that oppose natural transdifferentiation. To our knowledge, this is the first demonstration that a naturally occurring cell-fate conversion is actively restrained by identity-safeguarding chromatin regulators analogous to those previously described as barriers to experimentally-induced reprogramming. Together with the identification of LIN-15A, LIN-56 and multiple SynMuvB factors as new regulators of Y-to-PDA transdifferentiation, these findings support a model in which successful cell-fate conversion requires both instructive plasticity-promoting factors and the alleviation, by factors we termed Licensers, of chromatin-based constraints that stabilize differentiated cell identities. As stated by reviewer #2, " This is a well-executed genetic study that […] introduces a useful conceptual distinction between Driver and Licenser factors"

Point-by-point description of the revisions

This section is mandatory. Please insert a point-by-point reply describing the revisions that were already carried out and included in the transferred manuscript. I

Reviewer 1

- **As the authors acknowledge in the section at the end of the discussion (Limitations of this study) it is not established that LIN-15A has a cell-autonomous function in Y-to-PDA transdifferentiation. Given that LIN-15A has a cell non-autonomous function in vulval development (Herman and Hedgecock, 1990) it is possible that its function here could also be. The authors have used an egl-5 promoter to rescue lin-15A through expression in rectal cells; however, all these cells are in a neighborhood. The lack of a promoter that is specfic for Y has impeded answering this question (a standard genetic mosaic analysis would be problematic because of the incomplete penetrance of the mutation). Although this issue is addressed in the section at the end of the Discussion, I think most readers would like to see this**

acknowledged earlier in the presentation, perhaps after describing the egl-5 rescue experiment.

We thank the reviewer for this comment and agree that our data do not formally demonstrate a Y-cell autonomous role for LIN-15A during Y-to-PDA transdifferentiation, as we discussed in the manuscript. As suggested, we have modified the Results section immediately after the *egl-5* rescue experiment to explicitly acknowledge this limitation early-on (see p8, l168-170) and retained the discussion in the "Limitations of the study" section.

- **The experiment shown in Figure S1B is unconvincing. To show that they are detecting a LIN-15A-LIN-56 heterodimer, the authors need to show that antibody tags to both proteins detect the band. Mass spectrometry or biochemical purifications would also be helpful. As it is, they show the protein(s) detected depend genetically on lin-56 and lin-15A. It was also unclear what the other bands were in the mutant backgrounds**

We agree that the experiment shown in Figure S1B does not provide sufficient evidence to conclusively demonstrate the existence of a LIN-15A–LIN-56 heterodimer. While the detected species depend genetically on both *lin-15A* and *lin-56*, we agree that additional controls, such as detection through reciprocal tagging, biochemical purification, or mass spectrometry, would be required to firmly establish the molecular nature of the complex and to interpret the additional bands observed in the mutant backgrounds. As this experiment is not essential to the conclusions of the manuscript, we have removed Figure S1B and the associated statements from the revised version.

- **Lines 207-212. The authors are making an argument that LIN-15A and LIN-56 function as "Licensers" not "Drivers" because they are not strictly required but appear to facilitate the process. Could it be that LIN-15A and LIN-56 function as "Drivers" but that in their absence the fidelity of the process is compromise? There are many ways that genetic redundancy can be manifested at biochemical levels and the concern is that there are other interpretations of the data. In this regard, the authors should consider rewriting the Abstract to focus on the genetic results underpinning the work. The current version or the Abstract focuses on an interpretation of the data, not the data itself.**

We thank the reviewer for this thoughtful comment. We agree that the Driver/Licenser terminology represents an interpretation of the genetic data and that additional activities acting alongside LIN-15A may exist: *lin-15A* alleles used in this study correspond to null alleles - that is total loss of *lin-15A* activity - and approximately half of the animals still successfully undergo Y-to-PDA transdifferentiation in these null mutants. Thus, *lin-15A* activity is either not strictly required to facilitate the initiation of the process (e.g., threshold

model). Or this may point to other factors (than *lin-56*) able to somewhat compensate for *lin-15A* absence and that remain to be identified. In line with this interpretation, while we retrieved several alleles for some of the genes identified in our forward genetic screen (in which *lin-15A* was identified), that screen may not have been saturated. Note that both these hypotheses are compatible with a role for LIN-15A as a licenser of the initiation of the process.
Importantly, our distinction between "Drivers" and "Licensers" is not solely based on the incomplete penetrance of *lin-15A* and *lin-56* null mutants. First, the distinction reflects the different biological roles inferred from our genetic analyses. The previously characterized factors CEH-6, SOX-2, SEM-4, EGL-27, EGL-5 and HLH-16 are conserved plasticity factors that promote the initiation of transdifferentiation. Their loss results in a complete, or near-complete, failure of Y-to-PDA initiation, and they act within a common plasticity-promoting network. By contrast, LIN-15A and LIN-56 define a genetically distinct pathway. They are neither upstream nor downstream of the Driver cassette, and display additive interactions with partial Driver mutants. Second, loss of LIN-15A does not affect the fidelity or outcome of transdifferentiation. In all defective animals examined, the Y cell retains its normal position, morphology and rectal markers, indicating a failure to initiate the process rather than the production of an aberrant cell type. Third, the fact that a core Driver set is involved in different transdifferentiation events (ie Y-to-PDA and K-to-DVB) but not *lin-15A* or *lin-56* further argues against LIN-15A acting as a Driver. And finally, and most importantly, *lin-15A* and *lin-56* antagonize SynMuvB chromatin regulators known to safeguard differentiated cell identities, while the Drivers do not. In fact, the transdifferentiation process is mostly restored in some *lin-15A; SynMuvB* double null mutants, suggesting that LIN-15A main function is to block these genes activities. We therefore favor a model in which the Drivers cassette triggers transdifferentiation, whereas LIN-15A and LIN-56 facilitate the process by alleviating inhibitory constraints imposed by identity-safeguarding mechanisms. We have reformulated this in the manuscript in order to make it clearer and also clarified how we define Drivers and Licencers activities (see p10, l211-217; p13, l283-289 and p14 l312-315). We have further reformulated the abstract to integrate the reviewer's comments.

Minor Points

1. Line 279. The authors state that "LIN-15A becomes dispensable when member of the SynMuvB factors are absent." This statement is not completely accurate as the suppression is incomplete. – Addressed, the statement has been reworded in the revised version (see p13, l285-286)

2. Line 294. The number in Tagble S1 is 58.8% not 65% – Addressed, thank you for spotting this, Table S1 was correct, and the typo in the Results section was corrected (see p15, l319).

3**. Lines 300-301. I couldn't find the data for lin-40. –** The data can be found in Fig. 3Bii (which we have more clearly indicated in the text) and SI table 1.

4. Line 363. Should be "represses cell cycle genes." - Addressed

5. Line 862. AJM-1 is not a tight junction component. AJM-1 is best described as a component of apical junctions. – Absolutely ! Addressed

Reviewer 2

- **The conclusions derived from the presented data are generally comprehensible but should be phrased more carefully to grant full legitimacy. The reason is that the central mechanistic claim that LIN-15A licenses Td by antagonizing most of the SynMuvBs chromatin factors, including DREAM, rests on whole-animal ChIP-seq that cannot resolve the Y cell. The authors acknowledge that "it was not technically feasible to purify sufficient Y cells for analysis" and therefore use synchronized unstarved L1 whole-animal lysates. This is certainly legitimate, but demands more tact when using such a conclusion as the headline claim.**

  We thank the reviewer for this important comment and agree that the mechanistic conclusions drawn from the ChIP-seq data should be presented more cautiously. As noted by the referee and in the manuscript, it was not technically feasible to isolate sufficient Y cells for chromatin profiling and therefore all ChIP-seq experiments were performed on synchronized whole-animal L1 populations. We agree that these experiments cannot directly establish the mechanism operating in the Y cell. Rather, our genetic analyses demonstrate that LIN-15A antagonizes identity-safeguarding SynMuvB factors during Y-to-PDA transdifferentiation. The ChIP-seq data provide an additional and independent line of evidence suggesting that this antagonism may involve modulation of DREAM chromatin occupancy. We have rephrased to state this more clearly. We thus have revised the Abstract (see p2, l7), Introduction (p6, L110-112), Results (see p18-19, l405-420) and Discussion (see p23-24, l523-542 and p25 l566-575) to more clearly separate the conclusions supported by the genetic analyses from the mechanistic interpretation suggested by the ChIP-seq data. We further clarify that the relevance of this mechanism to the Y cell remains a hypothesis consistent with, but not directly demonstrated by, the available data.

- **Also, in the context of the ChIP-Seq experiments, it is understandable that it could not be conducted in a cell-specific manner, but two duplicates in some ChIP-Seq experiments (as stated in the material and methods) is below standard.**

  We thank the reviewer for this comment and agree that two biological replicates represent the lower end of what is generally desirable for ChIP-seq analyses. To clarify, more biological samples were initially generated than are represented in the final analysis. In total, five independent biological preparations were performed for each genotype. However, the experimental design imposed substantial technical constraints. Because the experiments required tightly synchronized fed L1 populations (ie, not using a starvation

step), standard synchronization procedures could not be used and animals instead had to be collected through successive hatch pulses, resulting in considerably lower yields. Combined with the mutant backgrounds analyzed, this led to variable ChIP-seq quality across preparations. To ensure robustness, we restricted the final analyses to datasets that passed all predefined quality-control criteria. As a result, some conditions were ultimately represented by only two high-quality biological replicates. We agree that this limitation should be made more explicit and have added this information in the Materials and Methods section (p35 l774-779). Despite the reduced number of replicates retained for some conditions, the genome-wide binding patterns observed for LIN-15B and LIN-35 in wild-type animals closely recapitulated those reported previously by the Ahringer laboratory (Gal et al., 2022; SI table 2), supporting the overall robustness and biological validity of the datasets used in this study. More generally, we have also tempered the interpretation of the ChIP-seq experiments throughout the manuscript. We view these data as supportive evidence consistent with a chromatin-level mechanism, rather than as definitive mechanistic proof, and have revised the text to reflect this more clearly.

- **Regarding the genetic interactions with met-2: as MET-2 works in concert with other SET domain proteins, such as SET-25, and also HPL-2, is there a possibility they may be implicated?**

  We also considered the possibility that the interaction observed with MET-2 could reflect a broader involvement of the H3K9 methylation machinery, given the well-established functional relationships between MET-2 and other SET domain proteins. To address this possibility, we tested whether SET-25 and SET-32 losses suppressed the *lin-15A* phenotype. In contrast to *met-2* loss-of-function, neither *set-25* nor *set-32* mutations modified the transdifferentiation defects observed in *lin-15A* mutants. These observations suggest that the interaction is not a general property of all MET-2-associated SET domain proteins and may instead reflect a more specific role for MET-2 in this context, although we have not tested triple mutant combinations, such as *met-2; set-25; lin-15A* or *met-2; set-32; lin-15A*, and therefore cannot exclude additional contributions from these factors. However, based on the available genetic evidence, our data support a model in which the phenotype is more closely linked to the SynMuvB-centered identity-safeguarding machinery than to the canonical MET-2/SET pathways. We now mention these negative results p14, l290-295 and in the discussion (p22, l510-511) of the revised manuscript. HPL-2 itself was tested alongside the other SynMuvBs, as previously reported to be a SynMuvB (Fig. 4Ci). Loss of HPL-2 had the same effect than loss of the other SynMuvBs. Together these data further suggest that the canonical SynMuvB machinery is at play, including MET-2, but not a generic requirement for all H3K9 methyltransferases, and instead points toward a more specific role of MET-2 within the SynMuvB.

- **The fact that Y-to-PDA in males (which involves a cell division) shows the same lin-15A dependence as in hermaphrodites is informative and a bit underplayed. Since this argues against a cell-cycle-coupled mechanism (an important aspect of the reprogramming field) for LIN-15A, it is worth elaborating on this in the discussion.**

We thank the reviewer for this insightful comment and agree that this result deserves further discussion. One of our initial hypotheses was indeed that LIN-15A might be specifically required in transdifferentiation events that occur without a cell division. Cell division and DNA replication have long been proposed to facilitate cellular reprogramming by promoting the dilution or resetting of identity-safeguarding mechanisms. In this context, it was conceivable that LIN-15A and LIN-56 might compensate for the absence of such a process during hermaphrodite Y-to-PDA transdifferentiation. However, our data do not support this model. We found that LIN-15A and LIN-56 are similarly required for Y-to-PDA transdifferentiation in males, despite the fact that this event occurs through a cell division. Conversely, neither factor is required for the K-to-DVB transdifferentiation, which also occurs in the rectum at a similar developmental stage and likewise involves a cell division. Together, these observations argue that the requirement for LIN-15A is not determined by the presence or absence of cell division. Rather, they suggest that the Licensers activity is context-dependent and linked to specific cellular identities. We agree that this point also strengthens the notion that Licensers are distinct from Driver factors, which function in both Y-to-PDA and K-to-DVB transdifferentiation. We have therefore modified the discussion (see p20 l441-443 and l455-480).

Minor: all addressed

- in the legend of Figure 1 and other places, it should be "Fisher's exact" instead of "Fisher exact"
- line 31; exhibits instead of exhibit
- line 85: results instead of result
- line 228: involvement instead of involvment
- line 293: "of missing" in loss of lin-36 had no effect while loss ... lin-53 further
- lines 297 - 299: check sentence; reads not correct
- line 395: "with an increase"
- line 484: "with regard"

Reviewer 3

Based on the observation that LIN-15A does not affect SynMuvB expression in Y (figure S4), the authors conclude that antagonism of the SynMuvBs by LIN-15A is not likely mediated by a negative control of their expression, but rather by impacting their activity. However, as suggested by the authors, antagonistic functions on the same targe genes is also a possibility. The classical approach to test this would be through expression profiling. I understand that RNA-seq on single Y cells cannot be carried out for technical reasons and that bulk RNA-seq would not be informative. Importantly, the same reasoning applies to the ChIP-seq data that is presented in support for common regulatory functions of a subset of synMuvs and LIN-15A (Figure 6 and S6), which was obtained from whole animals. The relevance

of these results to the Y to PDA Td process is therefore extremely limited, as the claim that LIN-15A restricts lin-35/DREAM binding on a subset of target genes is based on a reported decrease in DREAM binding in lin-15 mutants in bulk chromatin. This is especially true as both DREAM and LIN-15A are widely expressed proteins.

We agree with the general limitation highlighted here. As the reviewer notes, neither expression profiling nor chromatin profiling can currently be performed specifically in the Y cell due to the extremely small number of cells involved and the lack of suitable purification strategies. Consequently, the ChIP-seq experiments were performed on synchronized – and fed - whole-animal L1 populations. These data do not directly establish the mechanism operating during Y-to-PDA transdifferentiation. Rather, our conclusions are based on two distinct observations. First, the genetic analyses demonstrate an antagonistic relationship between LIN-15A and multiple SynMuvB factors during transdifferentiation. Second, the ChIP-seq experiments provide independent evidence that LIN-15A can influence DREAM chromatin occupancy at the organismal level. We interpreted these observations together as supporting a model in which the genetic antagonism may involve modulation of SynMuvB/DREAM chromatin activity. We agree, however, that the ChIP-seq data do not demonstrate that these chromatin changes occur in the Y cell itself, nor do they identify the relevant target genes involved in Y-to-PDA transdifferentiation. We have therefore revised the manuscript to more clearly distinguish between the conclusions supported directly by the genetic analyses and the mechanistic interpretation suggested by the ChIP-seq experiments. Throughout the revised version, and in the discussion in particular, we present the chromatin-level model as a hypothesis consistent with the available data rather than as a demonstrated mechanism operating in Y (see p2, l7 ; p6, l110-112 ; p18-19, l405-420 ; p23-24, l523-542 and p25 l566-575).

In addition there are specific issues with Figure 6, which is mislabeled: upregulated and downregulated applies to gene expression, while the numbers refer to binding peaks. Why are some numbers in red (not mentioned in the legend). An example of the corresponding genome browser tracks should be shown in supplementary. Was a spike-in used to normalize data?

We thank the reviewer for these helpful suggestions. We agree that the terminology "upregulated" and "downregulated" is potentially confusing in the context of ChIP-seq peaks. In the revised manuscript, we have replaced these terms with "up-bound" and "down-bound" in Figure 6. Regarding the red numbers, these were originally highlighted to emphasise the relatively small number of peaks showing decreased occupancy in *lin-15A* mutants compared to the other genotypes analyzed. However, as this information was not explained in the legend and may be confusing to readers, we have removed the color coding in the revised figure. Following the reviewer's suggestion, we have also added representative genome browser tracks in the Figure S6E to illustrate the binding changes described in Figure 6. No exogenous spike-in controls were used in these experiments. The ChIP-seq workflow was intentionally designed to closely follow that used by Gal et al. (2022), to allow direct comparison with the published LIN-15B and LIN-35 datasets. However, several observations suggest that the patterns reported here are unlikely to result

from normalization artifacts alone. First, the genome-wide binding profiles obtained for LIN-15B and LIN-35 in wild-type animals closely recapitulate those reported previously, providing an independent validation of the overall quality of the datasets. In addition, the different mutant backgrounds exhibit distinct peak gain/loss profiles rather than a common directional shift that would be expected from a systematic technical bias. Nevertheless, we acknowledge the absence of spike-in controls as a limitation of the dataset and have clarified this point in the revised manuscript in the Material and Methods section (see p36 l84-805).

Overall the discussion is highly speculative and could be shortened and refocused on the actual findings reported. For example, the fact that GO terms associated LIN-15B targets are associated with membrane processes (mentioned above) is not sufficient to speculate that LIN-15A could increase the delaminating capacities of Y by alleviating SynMuvB repression of membrane process genes.

Our intention was to discuss possible mechanisms that could connect the observed genetic interactions to the cellular events underlying Y-to-PDA transdifferentiation. We fully agree that some of these interpretations, such as the impact of the DREAM/LIN-15A antagonisms on membrane remodeling, are purely speculative in nature. We have removed the following sentence : "*In brief, the role of the Licensers would be to provide a favorable chromatin context for cellular processes that favor/install a plastic state, possibly through the modulation of membrane processes as suggested by our ChIP-seq analyses (Fig. S6).* » and changed it to "In this framework, Td Licensers would facilitate transdifferentiation by alleviating identity-safeguarding chromatin states, thereby creating a permissive context for the Drivers to execute the Td program. », and have removed the paragraph describing Y delamination. More generally, we have substantially shortened and refocused the Discussion section to answer the referee's comment.

The classical definition of a licensing factor is a protein (or complex) that allows the start of DNA replication from a replication origin. In the field of reprogramming, the term "licenser" has been applied to pioneer factors which 'license' transcriptional reprogramming by accessing chromatin to initiate a series of events, including binding of additional, non-pioneer transcription factors and additional chromatin regulators. Here the authors apply the term 'Licensers' to LIN-15A and LIN-56 as factors that facilitate the Td process. This may lead to confusion (and implications) as to what these factors are actually doing.

We thank the reviewer for raising this point. We agree that the term "licensing" has been used in several biological contexts, including DNA replication and, more recently, cellular reprogramming, where it is often associated with pioneer factors that initiate chromatin remodeling and transcriptional changes. However, our use of the term "Licenser" is intended to describe a distinct functional concept emerging from the genetic analyses presented here. We introduced this terminology to distinguish a class of factors that facilitate transdifferentiation by alleviating identity-safeguarding mechanisms from the previously identified "Driver" factors that actively promote the cell-fate transition itself. In this framework, LIN-15A and LIN-56 are not proposed to act as pioneer factors or direct

initiators of transcriptional reprogramming. Rather, the genetic data support a role in creating a permissive context for transdifferentiation by antagonizing mechanisms that oppose cell-fate change. We agree, however, that this distinction was not sufficiently defined in the original manuscript and may lead to confusion. We have therefore revised the Results and Discussion to explicitly frame it in the context of transdifferentiation ("Td Licenser"), define what we mean, and to clearly distinguish this usage from previous applications of the term in DNA replication and reprogramming studies (for instance, see p10, l211-217; p13, l283-289 and p14 l312-315).

Minor comments:

Abstract: why are Drivers and Licensers in capitals? How is Driver defined? – We use capital letters to signal that these represent two conceptual categories. However, this could be changed if that impairs reading. Drivers are defined in this study as plasticity factors whose loss completely prevents Td initiation (see p10, l211-217 and p14 l312-315).

Figure Aii: no PDE, ajm-1::GFP positive Y cells. It is not clear how the Y cell is identified-isn't ajm-1 supposed to surround the cell? The difference between the top and bottom ajm-1:egl-5 panels is not clear to a non expert. LIN-26 panel is missing. The Y cell is identified by its location at the ventral-most position on the anterior side of the rectal slit. AJM-1 is a component of the apical junctions, hence it is expressed at the apical domain of the Y cell. The LIN-26 typo has been corrected, the marker used in this experiment is the rectal-specific gene *egl-5* which labels the nucleus of the Y cell.

2F color scheme : licensers are not in yellow but pink – Addressed : they now are yellow in the revised version

Fig S1: need to provide more details about experimental conditions for WB-stage, conditions (reducing agents?), nature of Q2015 antibody. In the absence of this information hard to substantiate claim of a LIN-15/LIN-56 heterodimer in the text – See answer to reviewer #1 : we agree that this experiment is dispensable for the results presented in this manuscript and adds more questions than useful information, and it has been removed from the revised version.

Line 130. What is the nature of the LIN-56 protein? This would be useful information – Addressed. We have indicated this early on in the introduction (p5, l95-96) and in the Results section (p7, l132-134). Note that little is known about LIN-56 except its association with LIN-15A in VPC specification and that is equally possesses a THAP-like C2CH motif.

Line 38 yielding – addressed

Line 48 identities suggested by Blau and Baltimore (1991). - Addressed
