# Supplementary material for: The SynMuvA lin-15A licenses natural transdifferentiation by antagonizing identity safeguarding mechanisms
Source: PLoS Genet. 2026 Sep 25;22(9):e1012290. doi: 10.1371/journal.pgen.1012290 (PMC13641710; doi:10.1371/journal.pgen.1012290)
Supplement: Peer review history

# Author comment: Author response to Decision Letter 0

**DOI:** 10.1371/journal.pgen.1012290.r001

20 Jul 2026

Attachment Submitted filename: 168095_1_rebuttal_3797481_th6m2y.pdf

# Peer review: Decision Letter 0

**DOI:** 10.1371/journal.pgen.1012290.r002

**Authors:** Nathalie Pujol, Fengwei Yu

17 Aug 2026

Dear Dr Jarriault,

We are pleased to inform you that your manuscript entitled "The SynMuvA lin-15A licenses natural transdifferentiation by antagonizing identity safeguarding mechanisms" has been editorially accepted for publication in PLOS Genetics. Congratulations!

Before your submission can be formally accepted and sent to production you will need to complete our formatting changes, which you will receive in a follow up email. Please be aware that it may take several days for you to receive this email; during this time no action is required by you. Please note: the accept date on your published article will reflect the date of this provisional acceptance, but your manuscript will not be scheduled for publication until the required changes have been made.

Once your paper is formally accepted, an uncorrected proof of your manuscript will be published online ahead of the final version, unless you’ve already opted out via the online submission form. If, for any reason, you do not want an earlier version of your manuscript published online or are unsure if you have already indicated as such, please let the journal staff know immediately at plosgenetics@plos.org.

In the meantime, please log into Editorial Manager at https://www.editorialmanager.com/pgenetics/, click the "Update My Information" link at the top of the page, and update your user information to ensure an efficient production and billing process. Note that PLOS requires an ORCID iD for all corresponding authors. Therefore, please ensure that you have an ORCID iD and that it is validated in Editorial Manager. To do this, go to ‘Update my Information’ (in the upper left-hand corner of the main menu), and click on the Fetch/Validate link next to the ORCID field.  This will take you to the ORCID site and allow you to create a new iD or authenticate a pre-existing iD in Editorial Manager.

If you have a press-related query, or would like to know about making your underlying data available (as you will be aware, this is required for publication), please see the end of this email. If your institution or institutions have a press office, please notify them about your upcoming article at this point, to enable them to help maximise its impact. Inform journal staff as soon as possible if you are preparing a press release for your article and need a publication date.

Thank you again for supporting open-access publishing; we are looking forward to publishing your work in PLOS Genetics!

Yours sincerely,

Nathalie Pujol

Academic Editor

PLOS Genetics

Fengwei Yu

Section Editor

PLOS Genetics

Aimée Dudley

Editor-in-Chief

PLOS Genetics

Anne Goriely

Editor-in-Chief

PLOS Genetics

www.plosgenetics.org

BlueSky: @plos.bsky.social

----------------------------------------------------

Comments from the reviewers (if applicable):

Minor modification:

Please edit the flow in the abstract from results to hypothesis and proofread the english "Null mutant suggests that, rather than driving the process, LIN-15A licenses its initiation by antagonizes several chromatin-modifying complexes known to safeguard differentiated cell identities" to for example "Our genetic analyses show that lin-15A acts in parallel to previously described plasticity factors and reveal an antagonistic relationship between LIN-15A and multiple SynMuvB factors during transdifferentiation. ChIP-seq experiments provide independent evidence that LIN-15A influences DREAM chromatin occupancy at the organismal level. Together, these findings suggest that, rather than driving the process, LIN-15A licenses its initiation by antagonizing chromatin-modifying complexes known to safeguard differentiated cell identities.

Reviewer's Responses to Questions

**Comments to the Authors:**

**Please note here if the review is uploaded as an attachment.**

Reviewer #1: I have no further comments or requests. The improvements in this revised version justify acceptance.

Reviewer #2: Using the natural occurring phenomena of rectal-to-neuronal Y-to-PDA transdifferentiation in

Caenorhabditis elegans, the authors identify the THAP domain gene lin-15A as a novel factor involved in this transdifferentiation process. They show that lin-15A acts in parallel to previously described plasticity factors to initiate transdifferentiation by antagonizing a subset of chromatin-modifying complexes of the synmuvB class. Based on their data, the authors propose that lin-15A acts as a "licenser", as opposed to their previously described "drivers", to facilitate the transdifferentiation process.

Technicall challenges do not allow the authors to directly test the proposed model in

which key plasticity factors function as "Drivers", while others factors like lin-15A act as "Licensers" to attenuate identity safeguarding mechanisms at the chromatin level. Nonetheless the work paves the way to the identification of downstream targets and testing of this model at the molecular level.

The manuscript is well-written, the data well executed and interpretednd, providing useful genetic insight on an interesting topic

**********

**Have all data underlying the figures and results presented in the manuscript been provided?**

Large-scale datasets should be made available via a public repository as described in the *PLOS Genetics*  data availability policy, and numerical data that underlies graphs or summary statistics should be provided in spreadsheet form as supporting information.

Reviewer #1: Yes

Reviewer #2: Yes

**********

PLOS authors have the option to publish the peer review history of their article (what does this mean?). If published, this will include your full peer review and any attached files.

If you choose “no”, your identity will remain anonymous but your review may still be made public.

**Do you want your identity to be public for this peer review?**  For information about this choice, including consent withdrawal, please see our Privacy Policy.

Reviewer #1: No

Reviewer #2: No

----------------------------------------------------

**Data Deposition**

If you have submitted a Research Article or Front Matter that has associated data that are not suitable for deposition in a subject-specific public repository (such as GenBank or ArrayExpress), one way to make that data available is to deposit it in the Dryad Digital Repository. As you may recall, we ask all authors to agree to make data available; this is one way to achieve that. A full list of recommended repositories can be found on our website.

The following link will take you to the Dryad record for your article, so you won't have to re‐enter its bibliographic information, and can upload your files directly:

http://datadryad.org/submit?journalID=pgenetics&manu=PGENETICS-D-26-00767

More information about depositing data in Dryad is available at http://www.datadryad.org/depositing. If you experience any difficulties in submitting your data, please contact help@datadryad.org for support.

Additionally, please be aware that our data availability policy requires that all numerical data underlying display items are included with the submission, and you will need to provide this before we can formally accept your manuscript, if not already present.

----------------------------------------------------

**Press Queries**

If you or your institution will be preparing press materials for this manuscript, or if you need to know your paper's publication date for media purposes, please inform the journal staff as soon as possible so that your submission can be scheduled accordingly. Your manuscript will remain under a strict press embargo until the publication date and time. This means an early version of your manuscript will not be published ahead of your final version. PLOS Genetics may also choose to issue a press release for your article. If there's anything the journal should know or you'd like more information, please get in touch via plosgenetics@plos.org.

# Editor report: Acceptance letter

**DOI:** 10.1371/journal.pgen.1012290.r003

**Authors:** Nathalie Pujol, Fengwei Yu

PGENETICS-D-26-00767

The SynMuvA lin-15A licenses natural transdifferentiation by antagonizing identity safeguarding mechanisms

Dear Dr Jarriault,

We are pleased to inform you that your manuscript entitled "The SynMuvA lin-15A licenses natural transdifferentiation by antagonizing identity safeguarding mechanisms" has been formally accepted for publication in PLOS Genetics! Your manuscript is now with our production department and you will be notified of the publication date in due course.

The corresponding author will soon be receiving a typeset proof for review, to ensure errors have not been introduced during production. Please review the PDF proof of your manuscript carefully, as this is the last chance to correct any errors. Please note that major changes, or those which affect the scientific understanding of the work, will likely cause delays to the publication date of your manuscript.

Soon after your final files are uploaded, unless you have opted out or your manuscript is a front-matter piece, the early version of your manuscript will be published online. The date of the early version will be your article's publication date. The final article will be published to the same URL, and all versions of the paper will be accessible to readers.

For Research Articles, you will receive an invoice from PLOS for your publication fee after your manuscript has reached the completed accept phase. If you receive an email requesting payment before acceptance or for any other service, this may be a phishing scheme. Learn how to identify phishing emails and protect your accounts at https://explore.plos.org/phishing.

Thank you again for supporting PLOS Genetics and open-access publishing. We are looking forward to publishing your work!

With kind regards,

Anitha Samidurai

PLOS Genetics

On behalf of:

The PLOS Genetics Team

Carlyle House, Carlyle Road, Cambridge CB4 3DN | United Kingdom

plosgenetics@plos.org | +44 (0) 1223-442823

plosgenetics.org | Twitter: @PLOSGenetics
